# Cytosolic pH Controls Fungal MAPK Signaling and Pathogenicity

**DOI:** 10.1128/mbio.00285-23

**Published:** 2023-03-02

**Authors:** Tânia R. Fernandes, Melani Mariscal, Antonio Serrano, David Segorbe, Teresa Fernández-Acero, Humberto Martín, David Turrà, Antonio Di Pietro

**Affiliations:** a Departamento de Genética, Campus de Excelencia Internacional Agroalimentario ceiA3, Universidad de Córdoba, Córdoba, Spain; b Departamento de Microbiología y Parasitología, Facultad de Farmacia, Instituto Ramón y Cajal de Investigaciones Sanitarias (IRYCIS), Universidad Complutense de Madrid, Madrid, Spain; Karlsruhe Institute of Technology (KIT)

**Keywords:** MAP kinases, chemotropism, pH homeostasis, pathogenicity, virulence regulation

## Abstract

Mitogen-activated protein kinases (MAPKs) regulate a variety of cellular processes in eukaryotes. In fungal pathogens, conserved MAPK pathways control key virulence functions such as infection-related development, invasive hyphal growth, or cell wall remodeling. Recent findings suggest that ambient pH acts as a key regulator of MAPK-mediated pathogenicity, but the underlying molecular events are unknown. Here, we found that in the fungal pathogen Fusarium oxysporum, pH controls another infection-related process, hyphal chemotropism. Using the ratiometric pH sensor pHluorin we show that fluctuations in cytosolic pH (pH_c_) induce rapid reprogramming of the three conserved MAPKs in F. oxysporum, and that this response is conserved in the fungal model organism Saccharomyces cerevisiae. Screening of a subset of S. cerevisiae mutants identified the sphingolipid-regulated AGC kinase Ypk1/2 as a key upstream component of pH_c_-modulated MAPK responses. We further show that acidification of the cytosol in F. oxysporum leads to an increase of the long-chain base (LCB) sphingolipid dihydrosphingosine (dhSph) and that exogenous addition of dhSph activates Mpk1 phosphorylation and chemotropic growth. Our results reveal a pivotal role of pH_c_ in the regulation of MAPK signaling and suggest new ways to target fungal growth and pathogenicity.

## INTRODUCTION

Mitogen-activated protein kinase (MAPK) cascades are conserved eukaryotic signaling pathways that regulate a plethora of cellular functions, including growth, differentiation, and stress responses. Fungi have three conserved MAPKs, which are orthologs of Fus3/Kss1, Slt2/Mpk1, and Hog1 from Saccharomyces cerevisiae, and at least two of these have important roles in fungal pathogenicity on plants (1). We previously showed that in Fusarium oxysporum, a soilborne ascomycete pathogen that causes vascular wilt disease in more than 150 crops (2), the Kss1 ortholog Fmk1 is essential for invasive hyphal growth and pathogenicity (3, 4). Furthermore, F. oxysporum uses the cell wall integrity (CWI) MAPK Mpk1 for hyphal chemotropism toward signals released by plant roots into the soil (5, 6). The roles of these two MAPKs in invasive growth and chemotropism, respectively, appear to be broadly conserved in fungus-plant interactions (7, 8).

Ambient pH affects a wide range of biological functions, including nutrient acquisition, intracellular signaling, and cell growth. Fungi have evolved intricate mechanisms for sensing and modifying the surrounding pH (9). In fungal pathogens, acidification or alkalinization of the host pH by secretion of organic acids or ammonia, respectively, can dramatically alter the course of infection (10, 11). We recently found that infection of tomato plants by F. oxysporum is significantly reduced upon acidification of the rhizosphere via secretion of gluconic acid by the bacterial endophyte Rahnella aquatilis (12). On the other hand, F. oxysporum secretes a functional homologue of the plant regulatory peptide Rapid Alkalinizing Factor (RALF) to induce host alkalinization and increase its virulence on tomato plants (13). Importantly, alkalinization triggers phosphorylation of Fmk1 and promotes invasive growth, providing a link between ambient pH and MAPK function (13, 14). However, it is currently unknown how pH controls MAPK signaling during fungal infection.

In contrast to ambient pH, which can fluctuate dramatically, cytosolic pH (pH_c_) is tightly controlled by an elaborate pH homeostatic system (15) and acts as a key regulator of growth (16), metabolism (17–19), and cell fate (20, 21). Here, we show that fluctuations in ambient pH or in pH_c_ lead to rapid reprogramming of MAPK activity in F. oxysporum and in the model fungus Saccharomyces cerevisiae. Extracellular acidification or pharmacological inhibition of the major plasma membrane H^+^-ATPase Pma1 triggered a marked decrease in pH_c_, which resulted in rapid activation of the CWI MAPK Mpk1/Slt2 that was dependent on the essential sphingolipid-responsive protein kinase Ypk1. We further show that pH_c_ acidification in F. oxysporum causes an increase in the ceramide long-chain sphingoid base dihydrosphingosine that triggers Mpk1 phosphorylation and hyphal chemotropism. Our results establish a previously unrecognized role of pH_c_ in the regulation of MAPK signaling and fungal pathogenicity.

## RESULTS

### Ambient pH controls infection-related development in F. oxysporum.

**FIG 1:**
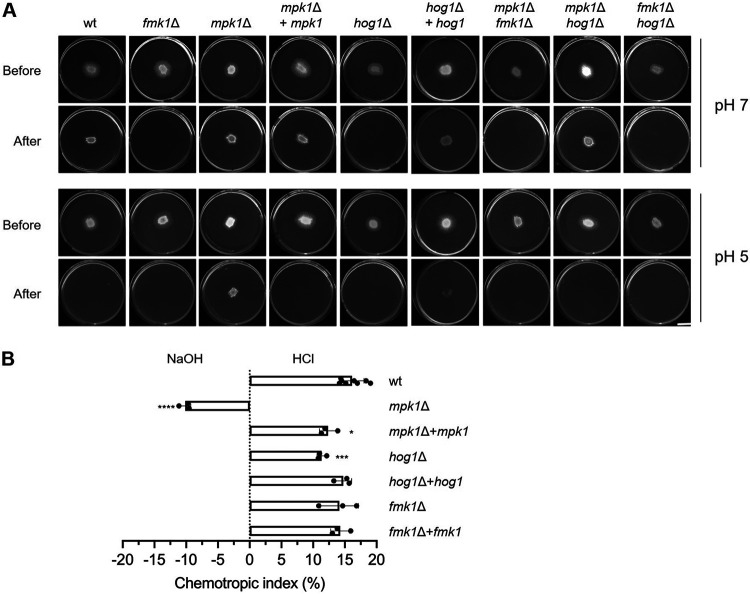
Differential role of MAPK cascades in pH control of fungal infection mechanisms. (A) Invasive growth of the F. oxysporum wild-type strain and the indicated single and double MAPK mutants was determined by spot-inoculating the indicated strains on top of a cellophane membrane placed on plates with potato dextrose agar (PDA) buffered at pH 7.0 or 5.0 with 100 mM MES. After 2 days at 28°C, plates were imaged (Before), the cellophane with the fungal colony was removed, and plates were incubated for an additional day to visualize the presence of mycelium that had penetrated through the cellophane (After). Images shown are representative of two independent experiments, each with 3 plates per treatment. Scale bar, 2 cm. (B) Directed growth of germ tubes of the F. oxysporum wild type and the indicated mutant strains was determined after 8 h exposure to opposing gradients of 25 mM HCl and NaOH. ****, *P* < 0.0001; ***, *P* < 0.001; *, *P* < 0.05 versus wild type (wt), according to Welch’s *t* test. Data show mean ± SD from at least three independent experiments (*n* = 500 germ tubes per experiment).

Previous work established that an increase of ambient pH promotes infection-related functions in F. oxysporum (14). Here, we set out to investigate the cellular mechanisms underlying pH-mediated control of fungal pathogenicity. Because alkalinization was previously shown to trigger rapid phosphorylation of the MAPK Fmk1, which is essential for invasive growth and plant infection (3, 14), we hypothesized that the effect of ambient pH in pathogenicity-related functions could be mediated by changes in MAPK activity. To test this, we first examined the role of the three known F. oxysporum MAPKs in invasive hyphal growth across cellophane membranes, a process that correlates directly with fungal pathogenicity on plants (4). Cellophane penetration assays conducted with the wild-type strain and all the possible combinations of single and double MAPK mutants (13) confirmed that, in line with a previous study (14), penetration by the wild-type strain is functional at pH 7.0 but not at pH 5.0 (Fig. 1A). These experiments also corroborated that invasive growth is strictly dependent on the MAPK Fmk1 since penetration was abolished in the single and double mutants lacking the *fmk1* gene. We further noted that cellophane crossing was also impaired in the *hog1*Δ mutant, which lacks the hyperosmotic stress response MAPK, but restored in the *mpk1*Δ*hog1*Δ double mutant, suggesting that Hog1 contributes positively to invasive growth, whereas Mpk1 has an inhibitory role. In line with this, the *mpk1*Δ mutant was able to cross the cellophane layer even at the restrictive pH 5.0, in contrast to the wild type and the *mpk1*Δ+*mpk1* complemented strain (Fig. 1A). Taken together, these results demonstrate that invasive growth of F. oxysporum is controlled by ambient pH and the concerted action of all three MAPKs; while Fmk1 is essential for the invasion process, Hog1 and Mpk1 act as positive and negative regulators, respectively.

We next examined the possible role of ambient pH in hyphal chemotropism, another infection-related process in F. oxysporum. Previous work showed that fungal germ tubes can redirect growth toward a chemoattractant gradient of peroxidase enzymes that are released by plant roots, and that this chemotropic response requires the Mpk1 MAPK pathway (5). Here, we found that F. oxysporum germlings exposed to competing gradients of alkaline and acidic pH grew preferentially toward the acid (Fig. 1B). Strikingly, deletion of Mpk1 led to inverted pH tropism, with the *mpk1*Δ mutant growing preferentially toward alkali, while in the *mpk1*Δ+*mpk1* complemented strain tropism toward acid was restored. We conclude that F. oxysporum hyphae can sense pH gradients and redirect growth toward acidic pH in a Mpk1-dependent manner.

### Shifts in ambient pH trigger rapid reprogramming of MAPK phosphorylation.

**FIG 2:**
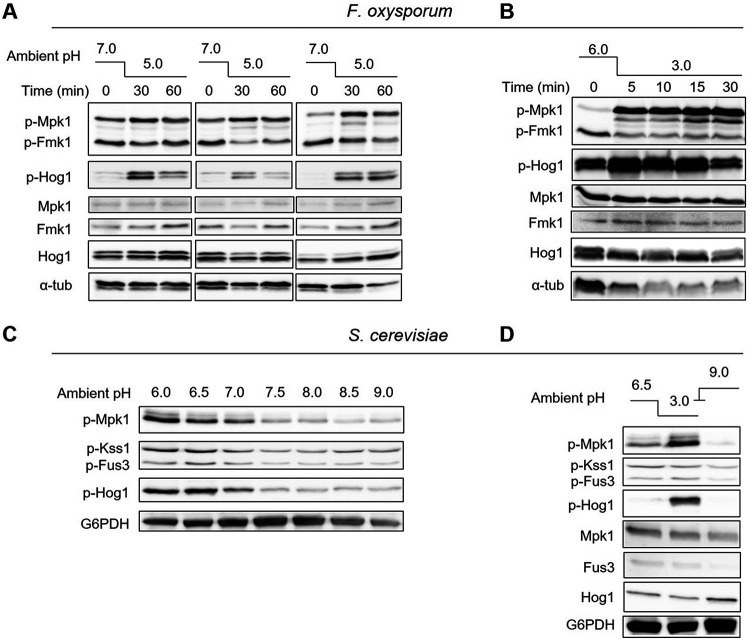
Shifts in ambient pH trigger rapid reprogramming of MAPK phosphorylation in F. oxysporum and S. cerevisiae. (A and B) F. oxysporum microconidia were germinated 15 h at 28°C, either in potato dextrose broth (PDB) buffered at pH 7.0 with 100 mM MES (A); or in yeast extract dextrose medium buffered at pH 7.4 with 20 mM HEPES (YD) and resuspended in KSU buffer at pH 6.0 (B), before the pH of the medium was shifted to 5.0 (A) or 3.0 (B) by adding diluted HCl. Total protein extracts collected at the indicated time points after the pH shift were subjected to immunoblot analysis with anti-phospho-p44/42 or anti-phospho-p38 MAPK antibody to specifically detect phosphorylated p-Mpk1 and p-Fmk1 or p-Hog1, respectively. Anti-Mpk1, anti-Fus3 and anti-Hog1 antibodies were used to detect total MAPK protein levels. Anti-α-tubulin (α-tub) was used as a loading control. The left panel shows immunoblots from 3 independent experiments. (C and D) S. cerevisiae cells grown overnight were either suspended in KSU buffer adjusted to the indicated pH values (C); or suspended in KSU buffer at pH 6.5 and preincubated 1 h at 30°C before shifting the pH of the growth medium to 3.0 or 9.0 by adding diluted HCl or NaOH, respectively (D). Protein extracts were collected 5 min after treatment and subjected to immunoblot analysis with anti-phospho-p44/42 or anti-phospho-p38 MAPK antibodies which specifically detect phosphorylated p-Mpk1 and p-Kss1/p-Fus3 or p-Hog1, respectively. Anti-Mpk1, anti-Fus3 and anti-Hog1 antibodies were used to detect total MAPK protein levels. Anti-G6PDH was used as a loading control.

The above findings suggested a possible link between pH and MAPK signaling in the control of infection-related functions. We previously observed rapid phosphorylation of Fmk1 upon extracellular alkalinization (14). Here, we found that extracellular acidification had the inverse effect, resulting in rapid dephosphorylation of Fmk1, concomitant with an increase in phosphorylation levels of the other two MAPKs Mpk1 and Hog1 (Fig. 2A and B). The timing and intensity of the response varied slightly between biological repeats and was dependent on the amplitude of the pH shift, but the trend was robust and reproducible across different experiments.

We next asked whether this response was conserved in other fungi. Using the model organism S. cerevisiae, we also observed rapid phosphorylation of Mpk1 and Hog1 upon extracellular acidification, whereas alkalinization had the opposite effect (Fig. 2C and D). To test whether activation of the CWI MAPK Mpk1 contributes to acid stress adaptation, we compared growth and survival of the F. oxysporum wild type and *mpk1*Δ strains under highly acidic conditions. Although conclusions for the *mpk1*Δ mutant are limited due to its generally reduced growth, we only detected very minor differences in acid resistance (Fig. S1). Taken together these findings suggest that ambient pH fluctuations trigger rapid changes in phosphorylation levels of the three fungal MAPKs.

10.1128/mbio.00285-23.1 FIG S1 The CWI MAPK Mpk1 has a minor role in adaptation to acidic pH. (A and B) Serial dilutions of fresh microconidia of the indicated strains were spot-inoculated on PDA plates adjusted to the indicated pH values by adding HCl (A) or supplemented with the indicated concentrations of acetic acid (AcOH) (B). Plates were incubated at 28°C in the dark and imaged after 3 days. Images shown are representative of two independent experiments with three plates each. Scale bar, 2 cm. (C) The percentage of cell survival of the F. oxysporum wild type (wt) and the *mpk1*Δ mutant after the indicated times of exposure to KSU buffer adjusted to pH 2 or 1 by adding HCl was measured by dilution plating and colony counting and normalized to time 0. *, *P* < 0.05 versus wt according to Welch’s t-test. Data show the mean ± SD of three replicate microwells. Download FIG S1, PDF file, 0.2 MB. Copyright © 2023 Fernandes et al. 2023 Fernandes et al. https://creativecommons.org/licenses/by/4.0/ This content is distributed under the terms of the Creative Commons Attribution 4.0 International license.

### pH-triggered changes in MAPK phosphorylation are mediated by fluctuations in pH_c_.

We next asked how ambient pH controls MAPK activity. We envisaged two possible scenarios that are not mutually exclusive: (i) changes in ambient pH directly or indirectly impinge on pH_c_, which in turn controls MAPK phosphorylation; and (ii) changes in ambient pH are sensed at the cell surface and directly transduced to the MAPK module.

**FIG 3:**
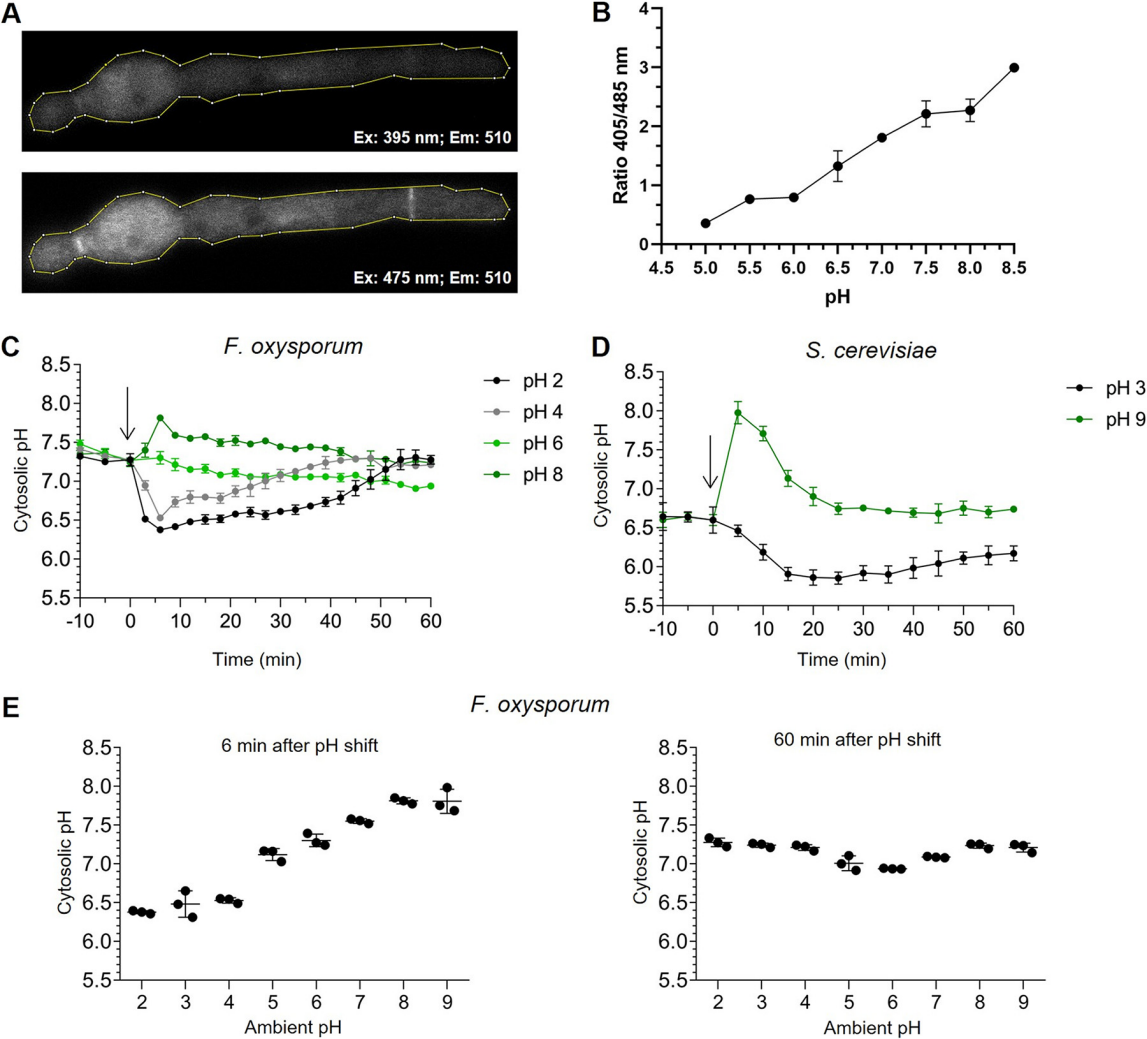
A shift in ambient pH triggers transient changes in cytosolic pH. (A and B) F. oxysporum microconidia germinated 15 h at 28°C in YD medium buffered at pH 7.4 with 20 mM HEPES or overnight in YPD medium were subjected to analysis of cytosolic pH (pH_c_) using a Zeiss LSM880 laser confocal microscope equipped with diode (405 nm) and Argon (488 nm) lasers, using a Plan Apo 63x oil 1.4 NA objective. A line delimiting the shape of the hypha was drawn (A), fluorescence intensity within the line was measured at 405 and 488 nm wavelength, and the 405/488 nm ratio was calculated for each pH value of the calibration curve (B). (C and D) The effect of ambient pH shifts on pH_c_ was measured in F. oxysporum (C) and S. cerevisiae (D) strains expressing the ratiometric pH probe pHluorin. F. oxysporum microconidia (C) or S. cerevisiae cells (D) were grown 15 h in YD or in yeast peptone dextrose (YPD) medium, respectively, resuspended in KSU buffer at pH 6.0 or 6.5, respectively, transferred to microwells and preincubated 50 min at 28°C or 30°C, respectively, before shifting the pH of the medium to the indicated values, as described in Fig. 2. pH_c_ was monitored spectrofluorometrically every 3 min starting 10 min before the pH shift. The ratio between the emission intensities at 510 nm after excitation at 395 nm and 475 nm was calculated and normalized to the standard curve (Fig. S2). Data show the mean ± SD of three replicate microwells from one representative experiment. Experiments were performed at least twice with similar results. (E) F. oxysporum microconidia were pretreated as described in panel A and pH_cyt_ was measured spectrofluorometrically 6 and 60 min after shifting the pH of the medium to the indicated values. Data show the mean ± SD of three replicate microwells from one representative experiment. Experiments were performed twice with similar results.

To follow pH_c_ in real time, we generated F. oxysporum strains expressing the genetically encoded pH-sensor pHluorin, a GFP-derivative that allows *in vivo* ratiometric pH_c_ measurement (22). A pHluorin-expressing transformant displaying high fluorescence levels (Fig. 3A) was subjected to *in vivo* calibration with buffers at different pH values, confirming the pH sensitivity and spectral characteristics of the expressed pHluorin protein (Fig. 3B). Independent measurements by confocal microscopy and spectrofluorometry in 96-well microtiter plates revealed a uniform distribution of pHluorin in the cytosol of F. oxysporum germlings, with a pH_c_ of 7.3 in our standard experimental conditions (Fig. 3A and C).

10.1128/mbio.00285-23.2 FIG S2 Pma1 inhibition by DES or membrane depolarization causes rapid acidification of pH_c_. (A and B) Pma1 inhibition by DES causes rapid and sustained acidification of pH_c_. F. oxysporum microconidia were pretreated as described in Fig. 3 before adding the indicated concentrations of DES to the medium. pH_c_ was monitored spectrofluorometrically (A) or by confocal microscopy (B) as described in Fig. 3C or (B), respectively. Data show the mean ± SD of three independent replicate microwells from one representative experiment. Experiments were performed twice with similar results. (C) Membrane depolarization by carbonyl cyanide p-trifluoromethoxyphenylhydrazone (FCCP) causes rapid and sustained acidification of pH_c_. F. oxysporum microconidia were pretreated as described in Fig. 3 before adding the indicated concentrations of FCCP to the medium. pH_c_ was monitored spectrofluorometrically as described in Fig. 3. Data show the mean ± SD of three independent replicate microwells from one representative experiment. Experiments were performed twice with similar results. (D) F. oxysporum microconidia were pretreated as described in Fig. 3, and 60 μM FCCP was added to the medium. Total protein extracts collected at the indicated times were subjected to immunoblot analysis with different antibodies as indicated in Fig. 2. Download FIG S2, PDF file, 0.3 MB. Copyright © 2023 Fernandes et al. 2023 Fernandes et al. https://creativecommons.org/licenses/by/4.0/ This content is distributed under the terms of the Creative Commons Attribution 4.0 International license.

We also generated transformants of S. cerevisiae strain BY4741 with a plasmid containing the *pHluorin2* gene driven by the *TEF1* promoter (21). Spectrofluorometric measurements of these transformants revealed a pH_c_ of around 6.6 (Fig. 3D), which is close to the value reported in previous studies (21).

We next asked whether pH_c_ is affected by changes in ambient pH. Acidification or alkalinization of the external medium triggered a marked down- or upshift, respectively, of pH_c_, both in F. oxysporum and S. cerevisiae (Fig. 3C and D). The most extreme down- or upshifts of external pH tested in F. oxysporum (from pH 6.0 to pH 2.0 or to pH 9.0) led to a fall or rise of pH_c_ of approximately 1.0 unit (from 7.3 to 6.4) or 0.5 units (from 7.3 to 7.8), respectively (Fig. 3E). The pH_c_ fluctuations were both rapid and transient, with a maximum amplitude around 6 min after the shift in ambient pH, followed by a gradual return to the homeostatic value of 7.3 (Fig. 3C to E). These findings suggest the existence of a robust pH homeostasis mechanism that protects the fungal cell during prolonged exposure to extreme ambient pH values.

**FIG 4:**
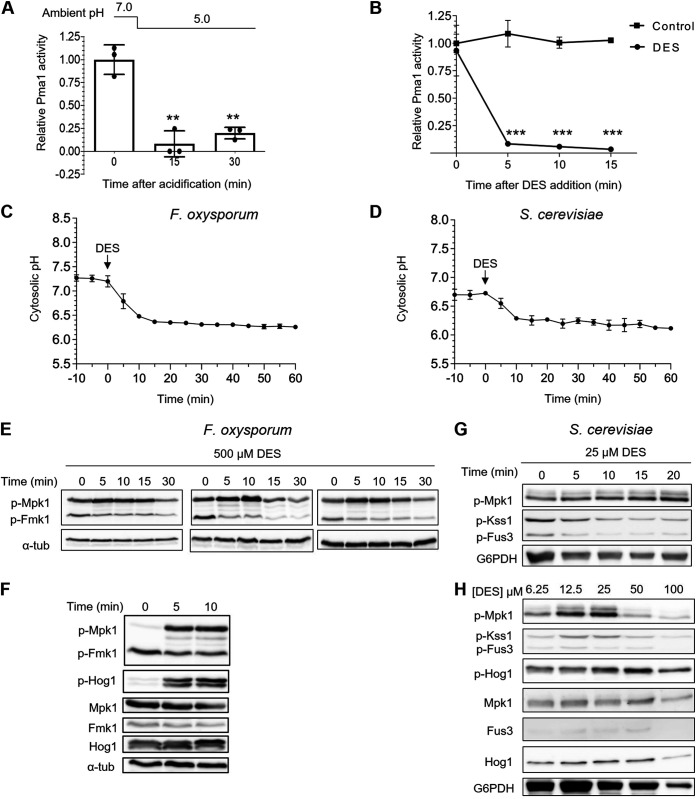
The plasma membrane H^+^-ATPase Pma1 regulates cytosolic pH and MAPK phosphorylation. (A and B) Activity of the major F. oxysporum plasma membrane H^+^-ATPase Pma1 is inhibited by acidic ambient pH and by the specific inhibitor diethylstilbestrol (DES). Microconidia of F. oxysporum were germinated as described in Fig. 2A before shifting the pH of the medium from 7 to 5 with diluted HCl (A) or adding 500 μM DES (B). Total membrane fraction was isolated from mycelia harvested at the indicated time points and H^+^-ATPase activity of Pma1 was measured and normalized to time zero. ***, *P* < 0.001; **, *P* < 0.01 according to Welch’s *t* test versus time zero (A) or untreated control (B). Data show the mean ± SD of three biological replicates from one representative experiment. Experiments were performed twice with similar results. (C and D) Pma1 inhibition by DES triggers rapid and sustained acidification of pH_c_. F. oxysporum microconidia (C) or S. cerevisiae cells (D) were pretreated as described in Fig. 3 before adding 500 or 25 μM DES, respectively. pH_c_ was monitored spectrofluorometrically every 5 min starting 10 min before DES addition. Data show the mean ± SD of three independent replicates from one representative experiment. Experiments were performed three times with similar results. (E–H) DES-triggered pH_c_ acidification leads to rapid Mpk1 phosphorylation and Fmk1 dephosphorylation. F. oxysporum microconidia (E and F) or S. cerevisiae cells (G and H) were subjected to DES treatment as described in panels C and D. Total protein extracts collected at the indicated times after addition of the indicated concentrations of DES were analyzed by immunoblot with different antibodies as indicated in Fig. 2. Panel E shows immunoblots from 3 independent biological experiments.

How do shifts in ambient pH lead to fluctuations in pH_c_? Previous studies identified the plasma membrane H^+^-ATPase Pma1 as a master regulator of pH_c_ in fungi (15). We noted that a downshift of external pH from 7.0 to 5.0 caused a rapid decrease of Pma1 H^+^-ATPase activity (Fig. 4A), suggesting a possible implication of Pma1 in the intracellular acidification observed in Fig. 3C. Furthermore, pharmacological inhibition of Pma1 H^+^-ATPase activity with the specific inhibitor diethylstilbestrol (DES) (23, 24) caused a rapid (5 min) and sustained drop in pH_c_ of approximately one pH unit, both in F. oxysporum (Fig. 4B and C; Fig. S2A) and in S. cerevisiae (Fig. 4D). The DES-induced pH_c_ acidification was independently confirmed by confocal microscopy measurements (Fig. S2B).

Pharmacological inhibition of Pma1 provides a powerful tool to manipulate pH_c_ independently of changes in extracellular pH. We therefore tested the effect of DES-triggered intracellular acidification on MAPK phosphorylation and detected a rapid increase in Mpk1 phosphorylation concomitant with a progressive dephosphorylation of Fmk1 (Fig. 4E and F). This response mimics that observed previously for extracellular acidification (Fig. 2A). We further found that treatment of F. oxysporum with the proton ionophore carbonyl cyanide-p-trifluoromethoxylphenylhydrazone (FCCP), which results in intracellular acidification, also induced rapid phosphorylation of Mpk1 concomitant with dephosphorylation of Fmk1 (Fig. S2C and D). Importantly, treatment with DES also triggered rapid phosphorylation of Mpk1 and dephosphorylation of the Fmk1 orthologs Kss1 and Fus3 in S. cerevisiae (Fig. 4G and H). We conclude that fluctuations in pH_c_ regulate MAPK phosphorylation through a mechanism that is broadly conserved in fungi.

### The Pal/Rim pathway has a minor role in pH-triggered MAPK regulation.

**FIG 5:**
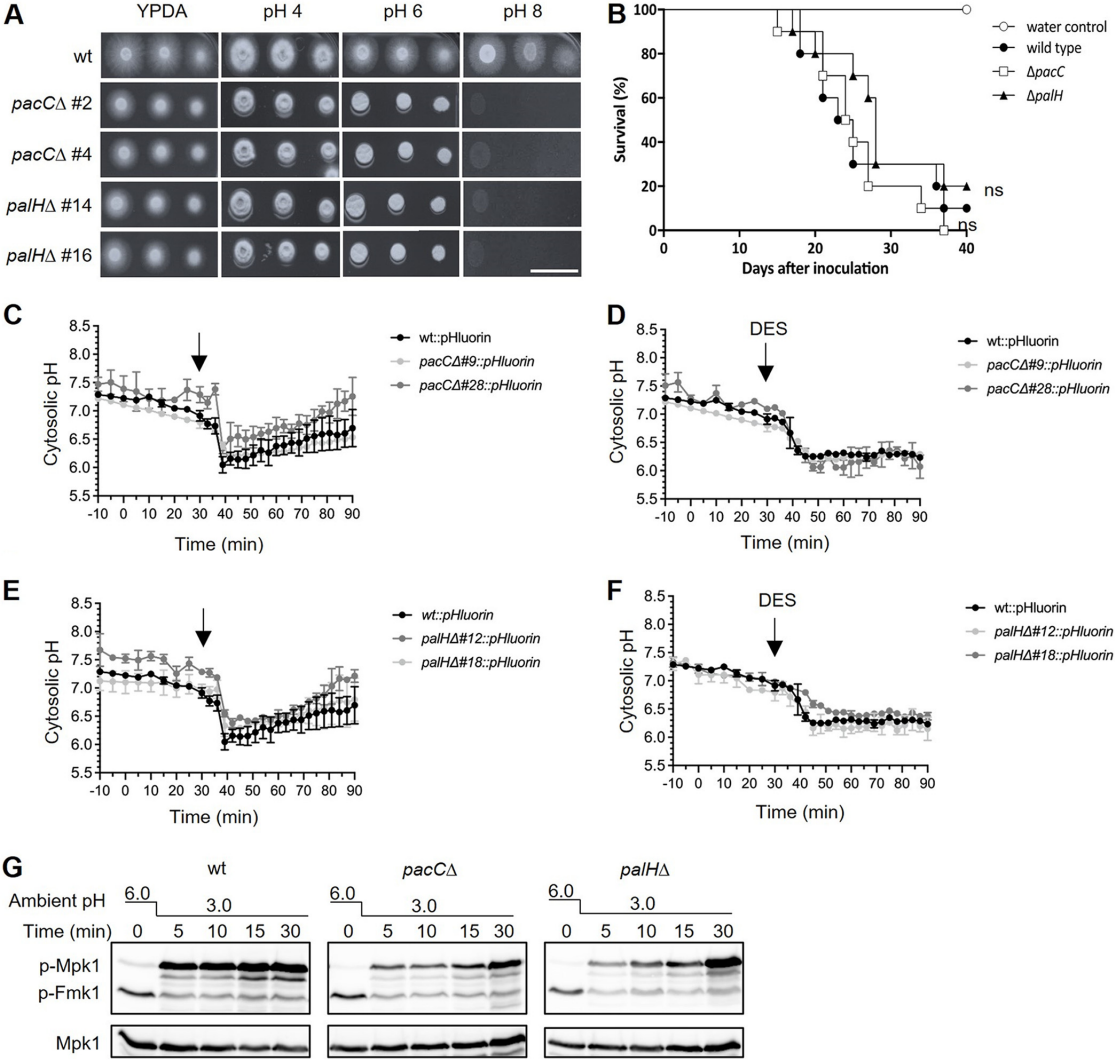
The Pal/PacC pathway is required for adaptation to high pH and contributes to acidification-triggered Mpk1 activation. (A) Serial dilutions of fresh microconidia of the indicated strains were spot-inoculated on plates containing Yeast extract Peptone Dextrose Agar (YPDA) medium buffered to the indicated pH with citrate-phosphate buffer. Plates were incubated at 28°C in the dark and imaged after 2 days. Images shown are representative of two independent experiments with three plates each. Scale bar, 2 cm. (B) Kaplan-Meier plot showing the survival of tomato plants inoculated with the wild-type strain or the indicated mutants. Groups of 10 plants were used. Data shown are from one representative experiment. Experiments were performed twice with similar results. ns = nonsignificant versus wild-type strain, according to log-rank test. (C–F) Microconidia of the indicated F. oxysporum strains were pretreated, as described in Fig. 2, before shifting the pH of the medium from 6.0 to 3.0 by adding diluted HCl (A and C) or adding 500 μM DES (B and D). pH_cyt_ was monitored spectrofluorometrically every 3 min. Data show the mean ± SD of three independent replicates from one representative experiment. Experiments were performed twice with similar results. (G) The indicated F. oxysporum strains were subjected to acidification of ambient pH as described in panel A. Total protein extracts collected at the indicated times after the pH shift were analyzed by immunoblot with anti-phospho-p44/42 to specifically detect phosphorylated p-Mpk1 and p-Fmk1. Anti-α-tubulin (α-tub) was used as a loading control.

To ask how pH_c_ controls MAPK activity, we first examined the Pal/Rim pathway, a broadly conserved mechanism of ambient pH sensing and response in fungi. Upon a shift to alkaline pH, the seven transmembrane domain receptor PalH/Rim21 initiates a signaling cascade resulting in proteolytic activation of the zinc finger transcription factor PacC/Rim101, that acts both as an activator of alkaline-expressed and a repressor of acidic-expressed genes (9, 25). To test the role of the Pal/Rim pathway in pH-mediated MAPK signaling of F. oxysporum, we generated *pacC*Δ and *palH*Δ deletion mutants both in the wild type and the pHluorin-expressing backgrounds (Fig. S3A to D). In line with previous reports (25, 26), the *pacC*Δ and *palH*Δ mutants exhibited a severe growth defect at high pH but were not affected in virulence on tomato plants (Fig. 5A and B). Moreover, pH_c_ dynamics of the *pacC*Δ and *palH*Δ mutants in response to external acidification or DES treatment was like that of the wild-type strain (Fig. 5C to F), although acid-induced Mpk1 phosphorylation was slightly delayed (Fig. 5G). Thus, the Pal/Rim pathway appears to have a minor role in pH-mediated MAPK regulation.

10.1128/mbio.00285-23.3 FIG S3 Targeted deletion of *pacC* and *palH* in F. oxysporum. (A and C) Schematic diagram showing targeted deletion of the F. oxysporum *pacC* (A) and *palH* (C) genes using the split-marker method. Gene knockout constructs were obtained by fusion PCR. Relative positions of restriction sites and Southern probes as well as of the PCR primers used are indicated. *hygR*, hygromycin resistance gene; P*gpdA*, *gpdA* promoter; T*trpC*, *trpC* terminator (both from A. nidulans). (B and D) Genomic DNA of independent transformants obtained in the wild type (wt, left panels) or the pHluorin-expressing background (right panels) was treated with *Nde*I (B) or *Xho*I (D), separated on 0.7% agarose gels, transferred to nylon membranes and hybridized with DIG labelled DNA probes from the indicated genes. Molecular weights of the hybridizing bands are indicated on the left. Download FIG S3, PDF file, 0.08 MB. Copyright © 2023 Fernandes et al. 2023 Fernandes et al. https://creativecommons.org/licenses/by/4.0/ This content is distributed under the terms of the Creative Commons Attribution 4.0 International license.

### The Pkh-Ypk branch mediates acid pH_c_-triggered Mpk1 activation upstream of the CWI MAPK cascade.

**FIG 6:**
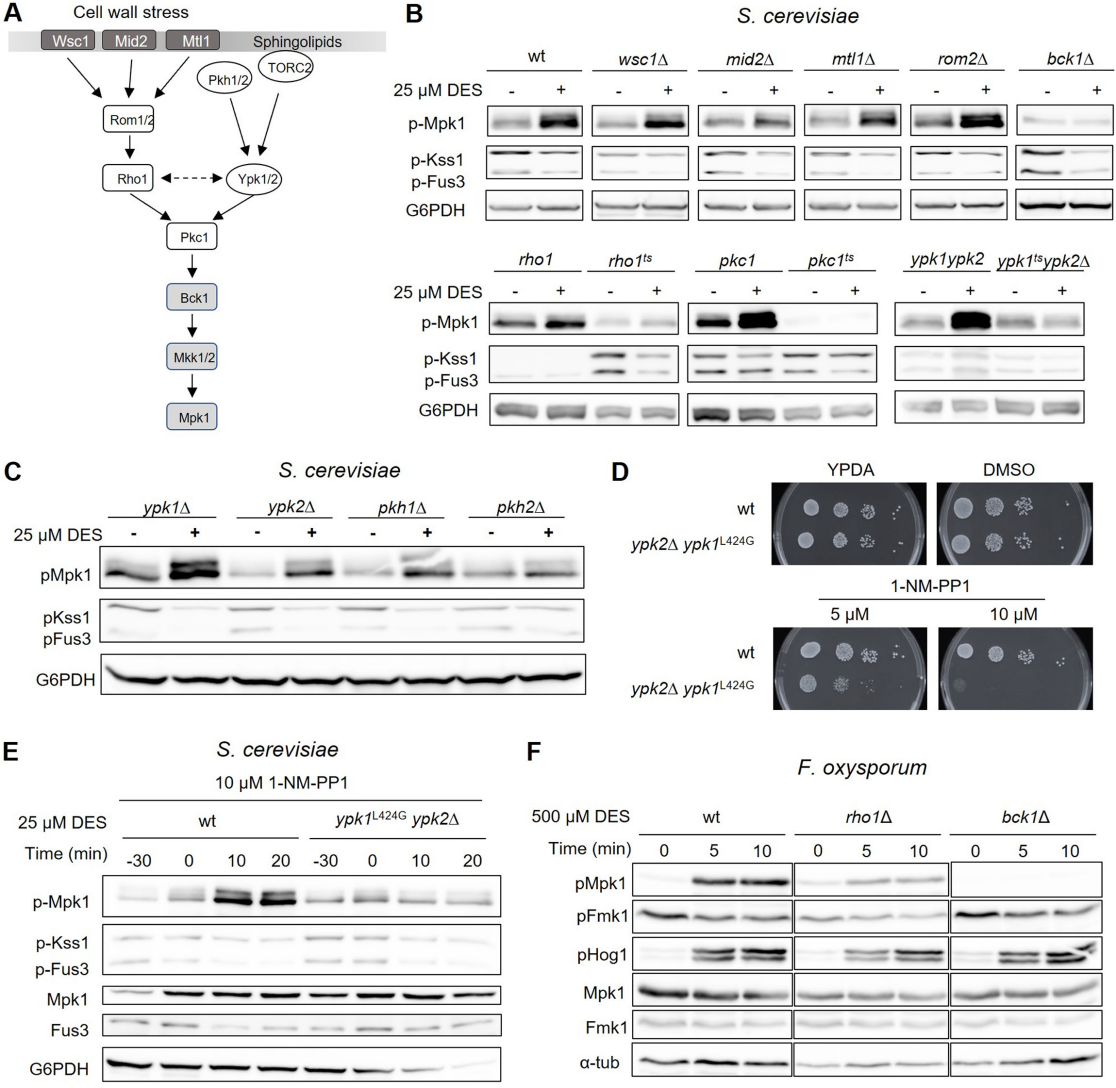
Acid pH-triggered activation of the CWI MAPK cascade is mediated by the Ypk1 sphingolipid signaling branch. (A) Schematic diagram of the CWI MAPK signaling pathway in S. cerevisiae. Modified from (Niles and Powers, 2014). (B, D, and E) Immunoblots showing MAPK phosphorylation in the wild type and in the indicated deletion or temperature-sensitive (ts) mutants of S. cerevisiae, at 0 (−) and 20 min (+) (B and D) or at the indicated times (E) after addition of 25 μM DES. In panel B, the strains were shifted to the restrictive temperature (34°C) for 60 min before DES addition. In panel E, 10 μM of the cell-permeable PP1 analog 1-NM-PP1 was added to the medium 30 min before DES addition. Total protein extracts were subjected to immunoblot with different antibodies as indicated in Fig. 2. (C) Serial dilutions of the indicated S. cerevisiae strains were spotted on YPDA medium supplemented or not with DMSO (solvent) or with the indicated concentrations of the specific Ypk1-AS inhibitor 1-NM-PP1. (F) Immunoblot showing MAPK phosphorylation in response to 500 μM DES in the wild type and in the indicated mutant strains of F. oxysporum. Protein extracts collected at the indicated time points were subjected to immunoblot with different antibodies as indicated in Fig. 2.

To gain insights into the mechanisms operating upstream of Mpk1, we performed Western blot analysis of DES-treated cells to screen a collection of S. cerevisiae mutants affected in known components of the CWI pathway (Fig. 6A) for defects in acid pH_c_-triggered MAPK responses. The deletion mutants in the cell surface sensors Wsc1, Mid2, or Mtl1, the downstream guanine exchange factor Rom2, or a *rho1* temperature-sensitive (ts) mutant were largely unaffected in DES-triggered Mpk1 phosphorylation and Fus3/Kss1 dephosphorylation (Fig. 6B). In contrast, mutants lacking the MAPKKK Bck1 or carrying a temperature sensitive allele of Pkc1 had constitutively low Mpk1 phosphorylation levels, although *bck1*Δ still exhibited a detectable dephosphorylation response of Fus3/Kss1. We next examined the role of the Pkh-Ypk upstream branch of the CWI MAPK cascade in DES-triggered MAPK regulation. In S. cerevisiae, the two AGC kinase paralogs Ypk1/2 are phosphorylated by the 3-phosphoinositide-dependent kinase 1 paralogs Pkh1/Pkh2 and the target of rapamycin complex 2 (TORC2) (27, 28) (Fig. 6A). The AGC kinase subfamily are serine/threonine kinases that were originally defined based on the sequence similarity of the catalytic domain found in PKA, PKG and PKC enzymes. Here, we found that single deletion mutants in *PKH1*, *PKH2*, *YPK1*, or *YPK2* genes were largely unaffected in DES-triggered Mpk1 phosphorylation and Kss1/Fus3 dephosphorylation, possibly due to functional redundancy of these gene paralogs (Fig. 6C). In contrast, an *ypk2*Δ *ypk1*-ts mutant, carrying a deletion of *ypk2* in a temperature sensitive *ypk1*-ts background failed to activate Mpk1 phosphorylation in response to DES-triggered pH_c_ acidification when shifted to the restrictive temperature previous to DES addition (Fig. 6B). Likewise, a *ypk2Δ ypk1^L424G^* mutant carrying a deletion of *ypk2* in an analog-sensitive (AS) *ypk1^L424G^* background (29) was defective in DES-triggered Mpk1 phosphorylation upon previous treatment with the PP1 analog 1-NM-PP1 (Fig. 6D and E). Together, these results indicate that acid pH_c_-triggered activation of Mpk1 in S. cerevisiae is mediated by the Pkh-Ypk branch upstream of the CWI cascade.

We next attempted to confirm these results in F. oxysporum. As in yeast, a partial Rho1 loss-of-function mutant of F. oxysporum (30) was largely unaffected in DES-triggered phosphorylation of Mpk1 and Hog1, while a mutant lacking the MAPKKK Bck1 exhibited constitutively low levels of Mpk1 phosphorylation but still showed rapid phosphorylation of Hog1 and dephosphorylation of Fmk1 (Fig. 6F). Repeated attempts to generate deletion mutants in the single F. oxysporum *ypk1* ortholog were unsuccessful (Fig. S4A to D), suggesting that Ypk1 is essential in F. oxysporum as reported in S. cerevisiae (31). We therefore recreated the analog-sensitive (AS) *ypk1^L424G^* allele used in S. cerevisiae (29) by changing the conserved leucine residue of F. oxysporum Ypk1 to glycine (*ypk1*^L368G^) (Fig. S5A). A transformant showing homologous replacement of *ypk1* with the *ypk1*^L368G^ allele was confirmed by Sanger sequencing (Fig. S5B). However, two independent monoconidial isolates of this transformant failed to show sensitivity to 1-NM-PP1 and therefore were not affected in DES-triggered Mpk1 phosphorylation (Fig. S5C and D). We next recreated the S. cerevisiae *ypk1*-ts allele (31) by changing the two conserved I^428^ and Y^480^ residues of F. oxysporum Ypk1 to T and C, respectively (Fig. S5E). However, in contrast to yeast, the growth of a F. oxysporum transformant carrying a homologous insertion of the *ypk1^I428T^*^,^*^Y480C^* allele was not significantly inhibited at high temperature, suggesting that these two mutations do not confer temperature sensitivity in this species (Fig. S5F and G). Taken together, these results suggest that the TORC2-Pkh-Ypk1 upstream module mediates activation of Mpk1 in response to pH_c_ acidification in S. cerevisiae, although this role remains to be functionally confirmed in F. oxysporum.

10.1128/mbio.00285-23.4 FIG S4 Failure to obtain *ypk1*Δ knockout mutants suggests that Ypk1 is essential in F. oxysporum. (A) Schematic diagram showing the targeted deletion of the F. oxysporum *ypk1* gene using the split-marker method. Gene knockout constructs were obtained by fusion PCR. Relative positions of restriction sites and Southern probes as well as of the PCR primers are indicated. Combinations of primers with the same colour were used for the PCR analyses in (C) and (D). *hygR*, hygromycin resistance gene; P*gpdA*, *gpdA* promoter; T*trpC*, *trpC* terminator (both from A. nidulans). (B and C) Genomic DNA of the wild type (wt) and independent hygromycin resistant transformants was subjected to PCR with the indicated pairs of primers (B) or treated with *Xho*I (C). The samples were separated on 0.7% agarose gels and imaged (B) or transferred to nylon membranes and hybridized with the DIG labelled DNA probe (C). Relative positions of the expected wild type or knockout (KO) hybridizing bands in (C) are indicated on the left. (D) Genomic DNA of the wild type (wt) and independent hygromycin resistant transformants was subjected to PCR with the indicated pairs of primers, separated on 0.7% agarose gels and imaged. M, Molecular size markers. Download FIG S4, PDF file, 0.2 MB. Copyright © 2023 Fernandes et al. 2023 Fernandes et al. https://creativecommons.org/licenses/by/4.0/ This content is distributed under the terms of the Creative Commons Attribution 4.0 International license.

10.1128/mbio.00285-23.5 FIG S5 Attempts of mutational analysis of *ypk1* in F. oxysporum. (A) Amino acid alignment showing the contextual conservation in F. oxysporum of the L424 residue of S. cerevisiae Ypk1, whose mutation to G in yeast causes sensitivity to the analog 1-NM-PP1 (Berchtold *et al*., 2012). (B) Screening of F. oxysporum transformants for the L368G mutation using RFLP analysis. Genomic DNA of the wild type (wt) and independent hygromycin resistant transformants was subjected to PCR followed by treatment with the restriction enzyme *Nar*I which cuts the DNA fragment carrying the *ypk1*^L368G^ mutation. As a positive control (+), PCR was performed on the DNA construct employed for transformation. (C) Serial dilutions of fresh microconidia of the wt and two monoconidial isolates of transformant no. 41 carrying the *ypk1*^L368G^ mutation were spot-inoculated on plates containing YPDA supplemented with the indicated concentrations of 1-NM-PP1. Plates were incubated at 28°C in the dark and imaged after 2 days. Images shown are representative of three independent biologic replicates. Scale bar, 2 cm. (D) Western blot showing MAPK phosphorylation in response to 500 μM DES in the wt and two monoconidial isolates of transformant no. 41 carrying the *ypk1*^L368G^ mutation. The specific Ypk1-AS inhibitor 1-NM-PP1 (40 μM) was added 60 min before DES addition (−60). Protein extracts were subjected to immunoblot analysis with anti-phospho-p44/42 MAPK antibody to detect phosphorylated p-Mpk1 and p-Fmk1. (E) Amino acid alignment showing the contextual conservation in F. oxysporum of the I484 and Y536 residues of S. cerevisiae Ypk1, whose simultaneous mutation to T and C, respectively, causes temperature sensitivity in yeast. (F) Analysis of temperature sensitivity in the F. oxysporum wild type strain and *ypk1-ts* no. 34 transformant carrying the I428T and Y480C mutations. Serial dilutions of fresh microconidia were spot-inoculated on PDA plates, incubated at indicated temperatures in the dark and imaged after 3 days. Images shown are representative of three independent biologic replicates. Scale bar, 2 cm. (G) Growth of the wt and the y*pk1*-ts no. 34 strain in PDB at 28°C or 37°C was monitored by measuring absorbance (Abs) at 600 nm. Values were normalized to time zero. Data show the mean ± SD of three independent replicates from one representative experiment. Experiments were performed twice with similar results. Download FIG S5, PDF file, 0.3 MB. Copyright © 2023 Fernandes et al. 2023 Fernandes et al. https://creativecommons.org/licenses/by/4.0/ This content is distributed under the terms of the Creative Commons Attribution 4.0 International license.

### Acidification of pH_c_ triggers changes in long-chain base membrane sphingolipids, which are relevant for Mpk1 activation and chemotropism of F. oxysporum.

**FIG 7:**
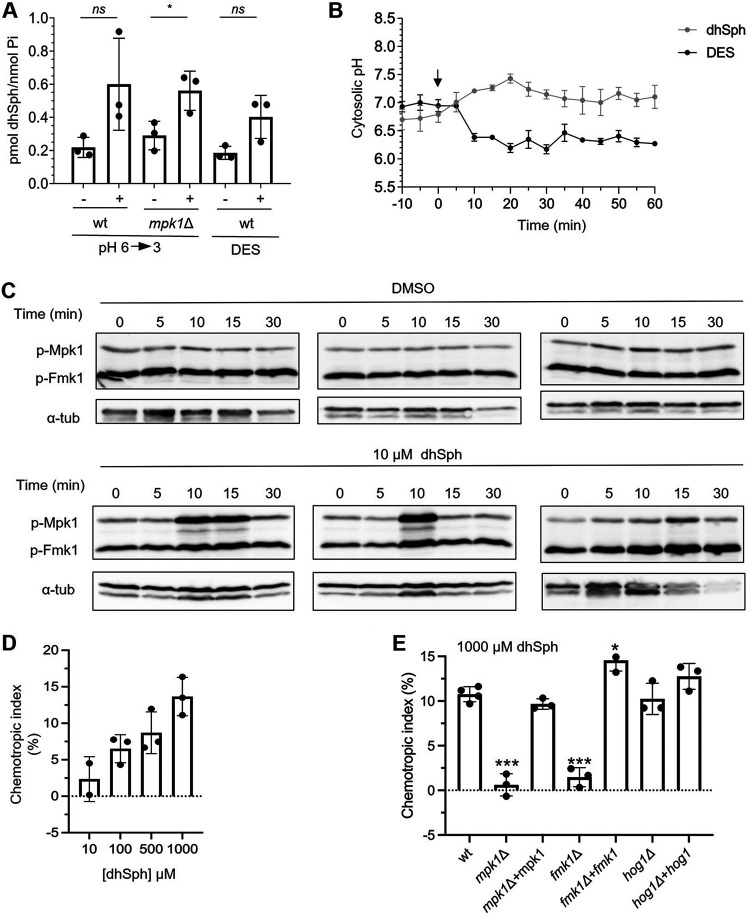
Dihydrosphingosine (dhSph) signals downstream of pH_cyt_ to regulate CWI MAPK signaling and hyphal chemotropism. (A) Acidification of ambient or cytosolic pH leads to increased levels of dhSph. F. oxysporum microconidia were pretreated, as described in Fig. 2, before shifting the pH of the medium from 6 to 3 with diluted HCl or adding 500 μM DES. Samples were collected before (−) or 10 min (+) after the treatment. Extracted lipids were analyzed by HPLC/MS-MS and the dhSph concentration was normalized to phosphate levels (Pi). Data show the mean ± SD of three independent biological experiments. *, *P* < 0.05 versus nontreated sample according to Welch’s *t* test. (B) Addition of dhSph does not affect pH_cyt_. F. oxysporum microconidia were pretreated, as described in Fig. 2, before adding either 500 μM DES or 100 μM dhSph to the medium. pH_cyt_ was monitored spectrofluorometrically starting 10 min before the treatment. Data show the mean ± SD of three independent replicate microwells from one representative experiment. Experiments were performed twice with similar results. (C) Western blot showing MAPK phosphorylation of F. oxysporum in response to addition of the solvent DMSO (upper panels) or 10 μM dhSph (lower panels). Total protein extracts collected at the indicated times were subjected to immunoblot analysis with anti-phospho-p44/42 MAPK to detect phosphorylated p-Mpk1 and p-Fmk1. Anti-α-tubulin (α-tub) was used as loading control. Immunoblots from 3 independent biological experiments are shown. (D and E) Directed growth of germ tubes of the F. oxysporum wild-type strain (D) or the indicated mutant strains (E) was determined after 8 h exposure to a gradient of the indicated concentrations of dhSph. ***, *P* < 0.001; *, *P* < 0.05 versus wt according to Welch’s *t* test. Data show mean ± SD of three independent biological experiments (*n* = 500 germ tubes per experiment).

In S. cerevisiae, the activity of Ypk1/2 is regulated by changes in the plasma membrane sphingolipid composition (29, 32, 33). We determined sphingolipid composition in F. oxysporum and found that a downshift of ambient pH or an acidification of pH_c_ by DES, led to an increase of the long-chain base (LCB) sphingolipid dihydrosphingosine (dhSph) (Fig. 7A, Fig. S6). An increase in the dhSph level was also observed in the *mpk1*Δ mutant, suggesting that this response is either independent or upstream of Mpk1. In line with the latter hypothesis, external application of dhSph triggered rapid phosphorylation of Mpk1 without affecting either pH_c_ or the phosphorylation status of the other MAPK Fmk1 (Fig. 7B and C). These findings suggest that activation of Mpk1 in response to pH_c_ acidification is mediated, at least in part, by an acid-triggered increase in the dhSph content.

10.1128/mbio.00285-23.6 FIG S6 Effect of extra- and intracellular acidification on sphingolipid composition in F. oxysporum. (A and B) Microconidia of the indicated F. oxysporum strains were pretreated as described in Fig. 3 before shifting the pH of the medium from 6 to 3 (A) or adding 500 μM DES (B). Samples were collected before (control) or 10 minutes after treatment. Extracted lipids were analyzed by HPLC/MS-MS and the concentration of each ceramide molecular species was normalized to total phosphate levels (Pi). Each graph shows the mean ± SD of three independent experiments. Download FIG S6, PDF file, 0.2 MB. Copyright © 2023 Fernandes et al. 2023 Fernandes et al. https://creativecommons.org/licenses/by/4.0/ This content is distributed under the terms of the Creative Commons Attribution 4.0 International license.

Mpk1 phosphorylation was shown to be triggered by plant chemoattractant signals (6) and is required for chemotropism of F. oxysporum toward tomato roots (5). Here, we found that dhSph not only activates Mpk1, but also functions as a chemoattractant of F. oxysporum hyphae. The chemotropic response to dhSph was dose-dependent and required both Mpk1 and Fmk1 (Fig. 7D and E). Collectively, these results suggest that dhSph acts as a signal downstream of cytosolic acidification to trigger the CWI MAPK signaling cascade and to induce hyphal chemotropism of F. oxysporum.

## DISCUSSION

Ambient pH sensing and MAPK cascades have long been known to act as key regulators of growth, development, and virulence in fungi, but the putative links between these two conserved signaling mechanisms have remained elusive (9, 11, 34). We previously found that extracellular alkalinization promotes invasive hyphal growth and plant infection in F. oxysporum (14) and that this effect is reversed upon acidification of the rhizosphere by organic acid-secreting bacteria (12). Here, we demonstrate a pivotal role of pH in hyphal chemotropism, an important infection-related process (5). F. oxysporum hyphae exposed to a pH gradient displayed robust tropism toward acid. Importantly, pH control of invasive growth and chemotropism is mediated by distinct MAPK signaling cascades. Alkaline-triggered invasive growth is activated via Fmk1 (3, 14), whereas chemotropism requires acid-mediated activation of Mpk1. Taken together, these findings reveal a finely tuned cooperation of different MAPK cascades during infection-related development of F. oxysporum, as previously reported for appressorial differentiation in the rice blast pathogen Magnaporthe oryzae (35).

Chemotaxis across pH gradients has been reported in a variety of organisms. Similar to F. oxysporum germ tubes, zoospores of the oomycete pathogen *Phytophthora palmivora* are attracted toward acidic pH (36). In contrast, African trypanosomes are attracted to alkali, a response that requires the cAMP/protein kinase A (PKA) pathway (37). An extracellular pH gradient was also shown to act as the dominant cue for the directional migration of MDA-MB-231 tumor cells during hematogenous metastasis (38). While the mechanism of chemosensing of pH gradients in eukaryotes remains largely unknown, it has been elucidated in several bacterial systems. For example, Escherichia coli exhibits bidirectional pH chemotaxis, allowing it to avoid extreme low and high pH environments, and this behavior is mediated by adaptive methylation of two major chemoreceptors (39). In the causal agent of stomach ulcer Helicobacter pylori, repulsion by acid and attraction toward alkali is important for virulence and requires at least two independent receptors capable of detecting acid gradients (40, 41). Further work is required to unravel the mechanisms underlying Mpk1-dependent sensing of a pH gradient by F. oxysporum hyphae.

### pH_c_ is a signal for regulation of MAPK activity and its downstream responses.

We found that the homeostatic pH_c_ in F. oxysporum hyphae is around 7.3, which is similar to that reported in Aspergillus niger (7.4 to 7.7) using either pHluorin (41) or ^31^P-NMR (42) and approximately one unit higher than the value of 6.5 measured in S. cerevisiae, both in this work and in previous studies (21). Whether this pH_c_ difference is related to the filamentous growth pattern of Fusarium and Aspergillus compared to the unicellular lifestyle of budding yeast remains to be determined. Alternatively, the low pH_c_ of S. cerevisiae could represent a specific adaptation to its specialized ecological niche. Testing of these two hypotheses will require further comparisons of pH_c_ values across a wider range of filamentous versus nonfilamentous fungal species. For example, the nonfilamentous pathogenic yeast Candida glabrata has a pH_c_ around 7.0, which represents an intermediate value between those of Fusarium/Aspergillus and S. cerevisiae (43). Interestingly, studies in C. albicans based on laser microspectrofluorimetry or ^31^P-NMR suggested the presence of a pH_c_ gradient along the germ tubes (44, 45), although no evidence for a pH_c_ gradient was detected along hyphal tips of a pHluorin-expressing strain of A. niger (46).

We found that pH_c_ homeostasis in F. oxysporum responds robustly to abrupt changes in ambient pH. Extreme up- or downshifts of external pH in the range between pH 2 and 9 led to rapid and transient fluctuations in pH_c_, followed by a gradual return to the homeostatic value. The amplitude of the pH_c_ fluctuations, with down- and upshifts of 1.0 and 0.5 pH units, respectively, is remarkable and resembles that observed in a previous study in A. niger (46). Because of the tight control of pH_c_ in all organisms, even relatively small fluctuations can trigger dramatic cellular responses. For instance, the homeostatic pH_c_ in human cells is generally around 7.2, while it is only 0.3 to 0.5 pH units higher in transformed cells and only 0.3 to 0.4 units lower in cells that trigger apoptosis (47). Similarly, programmed cell death (PCD) in yeast induced by the antimicrobial protein lactoferrin was preceded by a transient pH_c_ acidification of only 0.3 pH units, whose inhibition prevented PCD, indicating that this small pH_c_ downshift is sufficient to act as a triggering signal (20).

We noted that extracellular acidification caused a marked downshift of pH_c_, followed by a rapid phosphorylation response of the two stress-responsive MAPKs Mpk1/Slt2 and Hog1. This response was conserved in F. oxysporum and S. cerevisiae and was concomitant with a dephosphorylation of the invasive growth MAPK Fmk1/Kss1 and the pheromone response MAPK Fus3, respectively. Cross talk between the two stress response MAPKs and the pheromone response MAPK has been reported in budding yeast, although the underlying molecular mechanisms are poorly understood (48). In a previous study, we found that extracellular alkalinization promotes invasive growth and virulence of F. oxysporum by triggering rapid phosphorylation of the MAPK Fmk1 (14). Interestingly, pseudofilamentous growth in S. cerevisiae, which is mechanistically related to invasive hyphal growth and requires the Fmk1 ortholog Kss1, is also regulated by changes in pH_c_ (49). This suggests a conserved role of pH_c_-mediated MAPK regulation in the morphogenetic processes that mediate fungal invasion of the underlying substrate.

Importantly, reprogramming of MAPK phosphorylation in F. oxysporum and S. cerevisiae by extracellular acidification was fully recapitulated in the absence of external pH changes, by pharmacological inhibition of the H^+^-ATPase Pma1 or membrane depolarization, both of which caused a rapid decrease of pH_c_. These results clearly establish pH_c_ as a key regulator of fungal MAPK activity and are in line with a study in S. cerevisiae showing that pH_c_ acts as a second messenger upstream of protein kinase A to regulate the metabolic switch between phospholipid metabolism and lipid storage (50). A rapid downshift of pH_c_ in response to acid stress was previously proposed to promote cell survival by triggering growth arrest (51). Other stresses, such as heat shock or cell wall stress, also caused transient intracellular acidification associated with increased stress resistance (52, 53). In animal cells, fluctuations in pH_c_ have been linked to developmental transitions and signaling responses. For instance, an intracellular acidification by approximately one pH unit was observed during recovery of developmentally arrested dauer larvae of Caenorhabditis elegans (54), while in human hippocampal neurons stimulation with *N*-methyl-d-aspartate (NMDA) caused intracellular acidification (55).

Our findings confirms the pivotal role of the conserved plasma membrane H^+^-ATPase Pma1 in fungal pH_c_ homeostasis as well as in cell signaling and development (15). In S. cerevisiae, omeprazole-mediated inhibition of Pma1 caused a decrease in pH_c_ and activation of the AMP-activated protein kinase Snf1, suggesting a central role for pH_c_ in the regulation of the cell metabolic program (56). Intriguingly, an unequal distribution of Pma1 between mother and daughter cells was shown to be the causal mechanism for pH_c_ asymmetry, with Pma1 accumulating in the aging mother cell while being largely absent from the nascent daughter cell (57). In the human pathogen C. albicans, expression of a truncated version of Pma1 led to altered cation responses, disrupted vacuolar morphology and reduced filamentation (58). Meanwhile, RNAi-mediated silencing of the *pma1* gene in the citrus pathogen *Penicillum digitatum* resulted in reduced cell growth and pathogenicity as well as in cell wall alterations (59). Collectively, these results support the role of pH_c_ as a homeostatic sensor that controls the balance between cell growth and stress tolerance (51).

### LCB sphingolipid signaling links pH_c_ acidification to Ypk1-mediated activation of the CWI MAPK cascade.

Our analysis of S. cerevisiae mutants for defects in DES-triggered Mpk1 activation identified the AGC kinases Ypk1/2 as key upstream components of the pH_c_-triggered MAPK response. Interestingly, Ypk1/2 was previously detected in a genetic screen for S. cerevisiae mutants with increased sensitivity to acetic acid (60). Moreover, acetic acid-induced Ypk1 phosphorylation via TORC2 was shown to contribute to cell survival (61).

Our finding that Ypk1 is essential in F. oxysporum is in line with those in S. cerevisiae, where simultaneous deletion of the paralogs Ypk1 and Ypk2 is lethal. In contrast, the gene knockout mutants in the single *ypk1* homologs of A. nidulans and A. fumigatus were still viable, although they displayed a drastically sick phenotype and a complete lack of conidiation which precluded their further maintenance and analysis (62, 63). Interestingly, a conditional Aspergillus mutant carrying a *ypk1* allele driven by a glucose-repressible promoter, when grown under repressive conditions, exhibited a strong defect in vegetative growth and germination as well as increased thermosensitivity and reduced glycosphingolipid (GSL) levels (62, 63). A similar strategy could be applied in future studies to dissect the role of *ypk1* in F. oxysporum.

**FIG 8:**
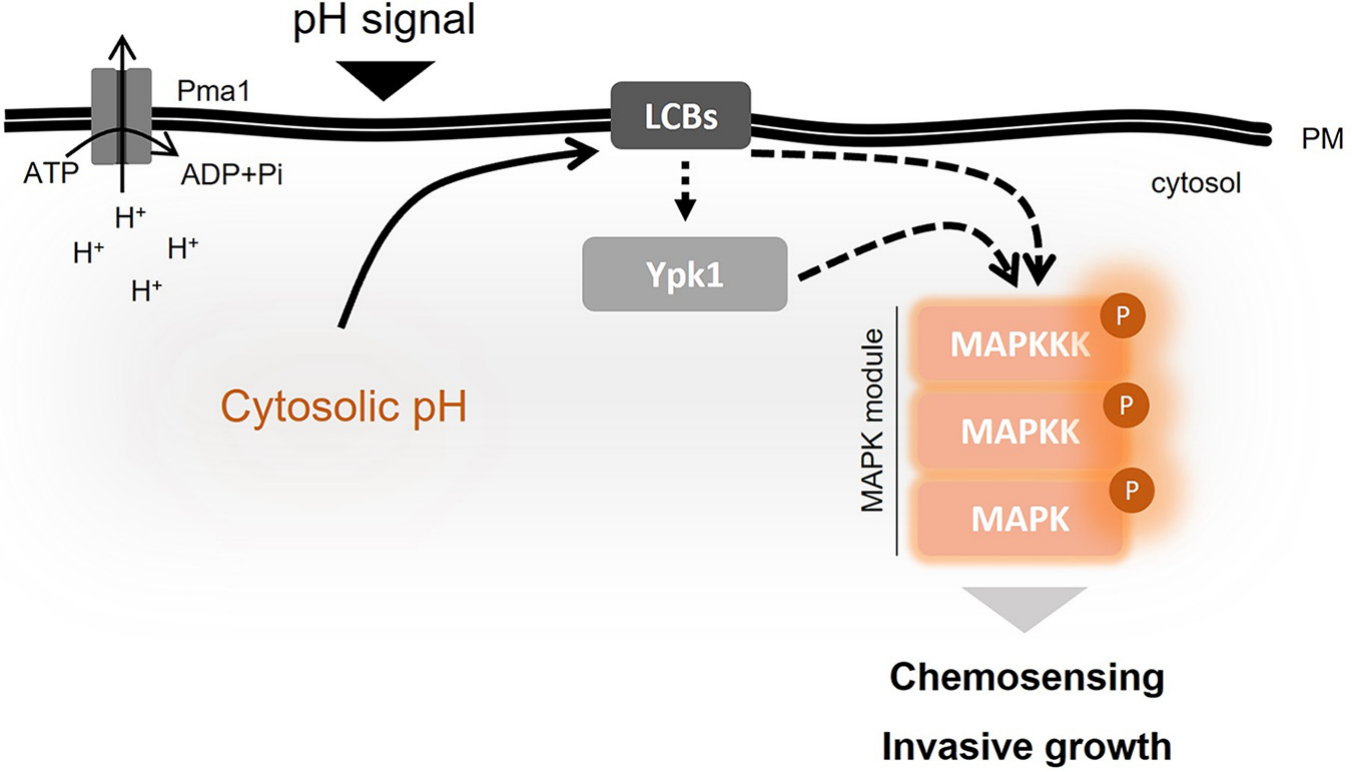
Cytosolic pH controls fungal MAPK signaling and pathogenicity-related functions. The plasma membrane H+-ATPase acts as a key regulator of pH_c_. Acidification of ambient or pH_c_ leads to increased levels of the membrane LCB dhSph, which in turn triggers activation of the CWI MAPK Mpk1 via the AGC kinase Ypk1. Acidification of pH_c_ also leads to inactivation of the invasive growth MAPK Fmk1 through an independent mechanism. The interplay of Mpk1 and Fmk1 regulates hyphal chemotropism, invasive growth, and virulence of F. oxysporum.

The LCB content in eukaryotic cells is usually much lower than that of the complex sphingolipids and ceramides, and the quantitative balance between these levels is tightly regulated. In S. cerevisiae there are two types of LCBs, phytosphingosine and dhSph (64). Here, pH_c_ acidification led to an Mpk1-independent increase in dhSph levels and exogenous addition of dhSph triggered rapid Mpk1 phosphorylation. This suggests that acid-triggered dhSph accumulation is an activating signal upstream of Ypk1 and the CWI MAPK cascade (Fig. 8). LCBs were previously shown to directly activate Pkh1 and Pkh2 in S. cerevisiae, leading to upregulation of Ypk1 and Ypk2 (65), while Ypk1 was found to connect sphingolipid biosynthesis with the CWI MAPK pathway (66, 67). On the other hand, TORC2-mediated phosphorylation of Ypk1 was shown to regulate sphingolipid biosynthesis in response to acetic acid stress (61). In S. cerevisiae, lipidomic profiling revealed dramatic changes in sphingolipid composition in response to acetic acid stress (68), while LCB levels were transiently increased during heat stress to cause G_0_/G_1_ arrest that was essential for thermotolerance (69).

Besides the acid stress response, other pH-regulated processes in F. oxysporum, such as hyphal chemotropism toward an acid pH gradient, could also be regulated by pH_c_-driven fluctuations in LCBs and downstream modulation of Ypk1/Mpk1. Indeed, we found that exogenously applied dhSph acts as a chemoattractant for F. oxysporum hyphae. Interestingly, TORC2-Ypk1 was shown to regulate actin polarization in S. cerevisiae via the Pkc1-Mpk1 MAPK cascade (28). Similarly, mTORC2 was shown to play a conserved role in regulation of the actin cytoskeleton of neutrophils during chemotactic migration (70).

A key finding of our work is that shifts in ambient pH cause dramatic changes in phosphorylation of the three conserved fungal MAPKs and that this response is mediated by fluctuations in pH_c_ and dhSph levels. We speculate that pH_c_ and LCB content may also act as regulators of alkalinization-induced MAPK responses. Intriguingly, the highly conserved PacC/Rim101 pathway, which regulates fungal adaptation to alkaline ambient pH, is also activated by alterations in plasma membrane lipids (71). The most upstream component of this pathway, the transmembrane sensor protein PalH/Rim21, was suggested to sense external alkalization through changes in plasma membrane lipid asymmetry by interacting with the inner membrane leaflet (72, 73). Changes in membrane lipid balance could thus play a general role in sensing both down- and upshifts of pH_c_. Furthermore, pH_c_ signaling could be mediated by additional mechanisms. In S. cerevisiae, cytoplasmic acidification was shown to induce a transition to a solid-like state, which was required for cell survival under conditions of nutrient starvation (74). Further investigations are needed to fully unravel the role of pH_c_ in fungal MAPK signaling, development and virulence.

## MATERIALS AND METHODS

### Fungal strains and culture conditions.

The tomato pathogenic isolate F. oxysporum f. sp. *lycopersici* Fol4287 (FGSC 9935) used throughout this study and the strains derived thereof are listed in Table S1. Standard methods for fungal growth, handling and genetic transformation were used as described previously (3, 75). For measurements of pH_c_ or H^+^-ATPase activity, sphingolipid profiling and Western blot analysis, freshly obtained F. oxysporum microconidia (5 × 10^6^ spores/mL) were germinated for 15 h at 28°C and 170 rpm in yeast extract-dextrose (YD) buffered at pH 7.4 with 20 mM HEPES (4-[2-hydroxyethyl]-1-piperazineethanesulfonic acid) unless indicated otherwise. Germlings were washed with potassium succinate urea (KSU) buffer (50 mM K_2_HPO_4_, 50 mM sodium succinate, 25 mM urea) adjusted to pH 6.0 with 1.2 M HCl and incubated for 60 min in the same buffer before initiating the experiment. External pH shifts were performed by adding diluted HCl or NaOH. For cell survival assays, samples of 5 × 10^7^ germlings were collected at the indicated time points, serially diluted, spotted on potato dextrose agar (PDA) plates, and CFU were counted after 48 h of incubation at 28°C.

10.1128/mbio.00285-23.7 TABLE S1 Fusarium oxysporum strains used in this study. Download Table S1, PDF file, 0.1 MB. Copyright © 2023 Fernandes et al. 2023 Fernandes et al. https://creativecommons.org/licenses/by/4.0/ This content is distributed under the terms of the Creative Commons Attribution 4.0 International license.

The S. cerevisiae wild type and mutant strains used in this study are listed in Table S2. Yeast cells were grown overnight in yeast peptone dextrose (YPD) medium at 30°C (25°C for temperature-sensitive strains) at 200 rpm, until reaching the exponential phase (optical density at 640 nm [OD_640nm_] of 0.9). Cells were then transferred to KSU buffer adjusted to pH 6.5 with 1.2 M HCl and incubated for 60 min at 30°C and 200 rpm before initiating the experiment. External pH shifts were performed by adding diluted HCl or NaOH. Temperature-sensitive strains were transferred to the restrictive temperature (34°C), as previously described (76).

10.1128/mbio.00285-23.8 TABLE S2 Saccharomyces cerevisiae strains used in this study. Download Table S2, PDF file, 0.1 MB. Copyright © 2023 Fernandes et al. 2023 Fernandes et al. https://creativecommons.org/licenses/by/4.0/ This content is distributed under the terms of the Creative Commons Attribution 4.0 International license.

### Western blot analysis of MAPK phosphorylation.

For Western blot analysis of MAPK phosphorylation, fungal samples were collected before (time zero) and at the indicated time points after the treatment. F. oxysporum samples containing 5 × 10^7^ germlings were collected at each time point and total protein was extracted in lysis buffer (0.2 M NaOH and 0.2% [vol/vol] β-mercaptoethanol), followed by precipitation with 7.5% (vol/vol) trichloroacetic acid (TCA) (77). Protein concentration was measured using the Bio-Rad DC protein assay kit (Bio-Rad, Alcobendas, Spain), using bovine serum albumin as standard. In addition, Coomassie blue staining was used for visual protein quantification. In S. cerevisiae, 25 mL aliquots of exponentially growing cells were collected from each time point and rapidly quenched by adding 2.0% (vol/vol) TCA. Protein extraction was performed as previously described (21) with modifications. Briefly, quenched samples were harvested by centrifugation, resuspended in 1 mL of 10 mM sodium azide, centrifuged again, and pellets were flash-frozen in liquid nitrogen and stored at −80°C. Frozen pellets resuspended in 0.5 mL of ice-cold TCA buffer (10 mM Tris pH 8.0, 10% [vol/vol] TCA, 25 mM NH_4_OAc, 1 mM Na_2_EDTA) were combined with one half volume of glass beads (0.5 mm diameter; Sigma-Aldrich, Madrid, Spain) and vortexed for 3 consecutive cycles of 90 sec interrupted by 30 sec intervals on ice. Precipitated protein was harvested by centrifugation at 13,400 rpm for 10 min at 4°C and resuspended in 75 μL resuspension buffer (100 mM Tris, 3% [vol/vol] SDS, pH 11.0). Protein concentration was measured using the Bio-Rad DC protein assay kit (Bio-Rad), using bovine serum albumin as standard.

Western blot analysis for determining MAPK phosphorylation status was performed as previously described (6). Briefly, protein samples were separated in 10% SDS-polyacrylamide gels and transferred to a nitrocellulose membrane using a Trans-Blot Turbo RTA midi nitrocellulose transfer kit (Bio-Rad). Phosphorylation of Mpk1 and Fmk1 MAPKs was detected using rabbit anti-phospho-p44/42 MAPK (Erk1/2) antibody (Thr202/Tyr204, no. 4370; Cell Signaling Technology, Danvers, MA). Phosphorylation of Hog1 MAPK was detected using rabbit anti-Phospho-p38 MAPK antibody (Thr180/Tyr182, no. 9211; Cell Signaling Technology). Total MAPK proteins were detected using a commercial mouse monoclonal anti-Mpk1 antibody (sc-165979, Santa Cruz Biotechnology, Heidelberg, Germany), a custom-designed polyclonal anti-Fus3/anti-Fmk1 antibody (SICGEN Research and Development in Biotechnology Ltd., Cantanhede, Portugal) based on amino acids 38 to 59 of the predicted F. oxysporum Fmk1 protein, or a commercial rabbit polyclonal anti-Hog1 antibody (sc-79079, Santa Cruz Biotechnology). Mouse anti-α-tubulin antibody (no. T9026, Sigma-Aldrich) and rabbit anti-glucose-6-phosphate dehydrogenase (G6PDH) (no. A9521, Sigma-Aldrich) were used as loading controls for F. oxysporum and S. cerevisiae, respectively. Hybridising bands were visualized using the ECL Select Western blotting detection reagent (GE Healthcare, Chicago, IL, USA) in a LAS-3000 detection system (Fujifilm España, Barcelona, Spain).

### Cytosolic pH measurements using the ratiometric fluorescent probe pHluorin.

A F. oxysporum strain expressing the pH-sensitive GFP variant pHluorin was obtained by cotransformation of fungal protoplasts with the hygromycin resistance cassette (*Hyg*^R^) (78) and a PCR fusion construct containing the *pHluorin* gene and the S. cerevisiae *adh5* terminator (22) fused to the strong constitutive Aspergillus nidulans *gpdA* promoter (78). Hygromycin resistant transformants were screened for the presence of the pHluorin expression cassette using PCR amplification with primers gpda15b and pHLNestRev and confirmed by fluorescence microscopy. Among the obtained transformants carrying the pHluorin cassette, the strain displaying the strongest intracellular fluorescence was selected for further studies. Measurements of pH_c_ in F. oxysporum were performed as previously described (79), either spectrofluorometrically in microtiter wells or by fluorescence confocal microscopy of single germlings. For spectrofluorometric measurements of pH_c_, germinated microconidia were transferred to KSU at pH 6.0 in 96-well microtiter plates and incubated for 30 min at 28°C before reading fluorescence intensities. Fluorescence emission at 510 nm after excitation at 395 nm and 475 nm was monitored over time in a TECAN spectrofluorometer (Infinite M200 PRO, TECAN Life Sciences, Switzerland). After subtracting the values of the pHluorin-negative wild-type background for each wavelength, the 395/475 nm ratio was calculated and converted to pH_c_ values using a pH calibration curve obtained with nigericin-permeabilized cells (79). Each experiment represents the average and standard deviation of three independent replicate wells. Experiments were performed at least twice.

For single-cell analysis of pH_c_, fluorescence intensities were recorded as previously described (79) using a Zeiss LSM880 laser confocal microscope equipped with diode (405 nm) and Argon (488 nm) lasers, using a Plan Apo 63x oil 1.4 NA objective. Images were set to 8 bits and the background was subtracted using the lookup table HiLo (Image/Fiji). Cell shape was delimited by drawing a line, the fluorescence intensity was measured within the line for each wavelength, and the 405/488 nm ratio was determined and converted to pH_c_ values using a pH calibration curve obtained with nigericin-permeabilized cells. Each experiment represents the average and standard deviation of at least three independent cells measured.

For measuring pH_c_ in S. cerevisiae, the wild-type strain BY4741 was transformed with the pYEplac181 plasmid (amp, LEU2) containing the *pHluorin* gene under the control of the *TEF1* promoter (21). Exponentially growing wild-type and pHluorin-expressing strains were resuspended in KSU pH 6.5, aliquoted into a 96-well microtiter plate and incubated for 30 min at 30°C before reading fluorescence intensities. Measurements and calculations of pH_c_ were performed as described above. Each experiment represents the average and standard deviation of three independent replicate wells. Experiments were performed at least twice.

### Determination of Pma1 H^+^-ATPase activity.

For determination of the activity of the plasma membrane H^+^-ATPase Pma1 in F. oxysporum, plasma membrane fractions were obtained as previously described (23) with minor modifications. Briefly, samples of 1.25 × 10^8^ germlings per time point were rapidly harvested by filtration through a nylon filter (mesh size 10 μm) and flash-frozen in liquid nitrogen. For crude membrane purification, mycelia were resuspended in 3 mL extraction buffer (0.3 M Tris-HCl pH 8.0, 0.3 M KCl, 30 mM EDTA, 5.3 mM dithiothreitol) supplemented with 40 μL protease inhibitor cocktail (Roche Life Sciences, Barcelona, Spain). Then, precooled 0.5-mm glass beads (5 mL per sample) were added and samples were vortexed for 3 consecutive cycles of 90 sec interrupted by 30 sec on ice. Cell lysates were centrifuged for 5 min at 1,157 *g*, and the supernatant was further centrifuged for 20 min at 18,472 *g*. Pellets were resuspended in a mixture of 100 μL glycerol buffer (20% [vol/vol] glycerol, 10 mM Tris-HCl pH 7.6, 1 mM EDTA, 1 mM DTT) and 900 μL of cold ultrapure water and centrifuged 30 min at 18,472 *g* to remove inorganic phosphate and other contaminants. Finally, the membrane fraction was resuspended in 100 μL glycerol buffer and the diethylstilbestrol (DES)-sensitive ATPase activity was measured as previously described (23), with minor changes. Briefly, samples with 6 μg membrane extracts were assayed for ATPase activity in a 96-well microtiter plate in the presence of 0.2 mM the Pma1-specific inhibitor DES or methanol (solvent control). The plate was incubated for 30 min at RT to allow irreversible inhibition of Pma1 activity by DES. Then, ATP-containing buffer (50 mM MES-Tris pH 5.7, 5 mM MgSO_4_, 50 mM KNO_3_, 5 mM sodium azide, 0.3 mM ammonium molybdate, 2 mM ATP) was added and samples were incubated for 40 min at 30°C. The reaction was stopped by adding detection buffer (2% [vol/vol] sulfuric acid, 0.5% [wt/vol] ammonium molybdate, 0.5% [wt/vol] SDS and 0.1% [wt/vol] ascorbic acid) and incubated for 20 min before reading absorbance at 750 nm in a TECAN spectrofluorometer. Specific Pma1 H^+^-ATPase activity was calculated by subtracting the residual activity value obtained in the presence of DES from the total activity (methanol), expressed in mmol/min/g protein assayed and normalized to time point zero for each time course. The results represent the average and standard deviation of three independent replicates. Experiments were performed at least twice.

### Quantification of hyphal chemotropism and invasive hyphal growth.

Chemotropic growth was measured using a quantitative plate assay described previously (5). Briefly, 10^6^ microconidia were embedded in 0.5% water agar, incubated 8 h at 28°C in the presence of a chemoattractant gradient, and the direction of germ tubes relative to a central scoring line was determined in an Olympus binocular microscope at 9, ×200 magnification. For each sample, five independent batches of cells (*n* = 100 cells per batch) were scored. Calculation of the chemotropic index and statistical analysis was done as described previously (5). Experiments were performed at least three times with similar results. For pH chemotropism, a gradient competition assay (5) was performed between two wells at both sides of the scoring line containing 25 mM HCl or NaOH, respectively, as chemoattractants.

Invasive hyphal growth through cellophane membranes was determined as previously described (4). Briefly, potato dextrose broth (PDB) agar plates buffered to pH 5 or pH 7 with 100 mM MES (2-[N-morpholino]ethanesulfonic acid) were covered with a cellophane membrane and 5 × 10^4^ microconidia were spot-inoculated on the top at the center of the plate. After 2 days of incubation at 28°C, the cellophane membrane with the fungal colony was carefully removed and plates were incubated for 1 additional day at 28°C. Plates were scanned before and after cellophane removal. Triplicates were performed for each strain and condition, and two independent experiments were performed with similar results.

### Sphingolipid profiling.

For quantitative analysis of sphingolipid species, samples of 5 × 10^7^ germlings were collected before (time zero) and at the indicated time points after the treatment. Samples were flash frozen in liquid nitrogen, lyophilized and submitted to quantitative sphingolipid analysis at the Lipidomics Shared Resource, Medical University of South Carolina, USA. The levels of the ceramide long-chain (sphingoid) bases (LCB) sphingosine (Sph) and dihydrosphingosine (dhSph), as well as sphingoid base-1-phosphates (S1P and dhS1P) and ceramide molecular species were measured by high-performance liquid chromatography/mass spectrometry (HPLC-MS/MS) (80). Quantitative analysis of sphingolipids was based on eight-point calibration curves generated for each target analyte. Synthetic standards along with a set of internal standards were spiked into an artificial matrix and then subjected to an identical extraction procedure to that of the biological samples. The extracted standards were analyzed by the HPLC/MS-MS operating in positive multiple reaction-monitoring (MRM) mode employing a gradient elution. Analyte-specific calibration curves were generated by plotting the analyte/internal standard peak area ratios against the analyte concentrations. Lipids with no authentic standards were quantitated using the calibration curve of their closest counterpart. The concentration of each sphingolipid species was normalized to the total phosphate level in each biological sample.

### Data availability.

All data needed to evaluate the conclusions in the paper are present in the paper and/or the supplementary files.

## References

[1] Turrà D, Segorbe D, Di Pietro A. 2014. Protein kinases in plant-pathogenic fungi: conserved regulators of infection. Annu Rev Phytopathol 52:267–288. doi:10.1146/annurev-phyto-102313-050143. 25090477

[2] Dean R, Van Kan JA, Pretorius ZA, Hammond-Kosack KE, Di Pietro A, Spanu PD, Rudd JJ, Dickman M, Kahmann R, Ellis J, Foster GD. 2012. The Top 10 fungal pathogens in molecular plant pathology. Mol Plant Pathol 13:414–430. doi:10.1111/j.1364-3703.2011.00783.x. 22471698 PMC6638784

[3] Di Pietro A, Garcia-Maceira FI, Meglecz E, Roncero MI. 2001. A MAP kinase of the vascular wilt fungus Fusarium oxysporum is essential for root penetration and pathogenesis. Mol Microbiol 39:1140–1152. doi:10.1111/j.1365-2958.2001.02307.x. 11251832

[4] López-Berges MS, Rispail N, Prados-Rosales RC, Di Pietro A. 2010. A nitrogen response pathway regulates virulence functions in Fusarium oxysporum via the protein kinase TOR and the bZIP protein MeaB. Plant Cell 22:2459–2475. doi:10.1105/tpc.110.075937. 20639450 PMC2929112

[5] Turrà D, El Ghalid M, Rossi F, Di Pietro A. 2015. Fungal pathogen uses sex pheromone receptor for chemotropic sensing of host plant signals. Nature 527:521–524. doi:10.1038/nature15516. 26503056

[6] Nordzieke DE, Fernandes TR, El Ghalid M, Turrà D, Di Pietro A. 2019. NADPH oxidase regulates chemotropic growth of the fungal pathogen Fusarium oxysporum towards the host plant. New Phytol 224:1600–1612. doi:10.1111/nph.16085. 31364172

[7] Sakulkoo W, Osés-Ruiz M, Oliveira Garcia E, Soanes DM, Littlejohn GR, Hacker C, Correia A, Valent B, Talbot NJ. 2018. A single fungal MAP kinase controls plant cell-to-cell invasion by the rice blast fungus. Science 359:1399–1403. doi:10.1126/science.aaq0892. 29567712

[8] Moreno-Ruiz D, Lichius A, Turrà D, Di Pietro A, Zeilinger S. 2020. Chemotropism assays for plant symbiosis and mycoparasitism related compound screening in Trichoderma atroviride. Front Microbiol 11:601251. doi:10.3389/fmicb.2020.601251. 33329491 PMC7729004

[9] Peñalva MA, Lucena-Agell D, Arst HNJ. 2014. Liaison alcaline: pals entice non-endosomal ESCRTs to the plasma membrane for pH signaling. Curr Opin Microbiol 22:49–59. doi:10.1016/j.mib.2014.09.005. 25460796

[10] Alkan N, Espeso EA, Prusky D. 2013. Virulence regulation of phytopathogenic fungi by pH. Antioxid Redox Signal 19:1012–1025. doi:10.1089/ars.2012.5062. 23249178

[11] Fernandes TR, Segorbe D, Prusky D, Di Pietro A. 2017. How Alkalinization drives fungal pathogenicity. PLoS Pathog 13:e1006621. doi:10.1371/journal.ppat.1006621. 29121119 PMC5679519

[12] Palmieri D, Vitale S, Lima G, Di Pietro A, Turrà D. 2020. A bacterial endophyte exploits chemotropism of a fungal pathogen for plant colonization. Nat Commun 11:5264. doi:10.1038/s41467-020-18994-5. 33067433 PMC7567819

[13] Segorbe D, Di Pietro A, Peréz-Nadales E, Turrà D. 2017. Three Fusarium oxysporum mitogen-activated protein kinases (MAPKs) have distinct and complementary roles in stress adaptation and cross-kingdom pathogenicity. Mol Plant Pathol 18:912–924. doi:10.1111/mpp.12446. 27301316 PMC6638227

[14] Masachis S, Segorbe D, Turrà D, Leon-Ruiz M, Furst U, El Ghalid M, Leonard G, López-Berges MS, Richards TA, Felix G, Di Pietro A. 2016. A fungal pathogen secretes plant alkalinizing peptides to increase infection. Nat Microbiol 1:16043. doi:10.1038/nmicrobiol.2016.43. 27572834

[15] Kane PM. 2016. Proton transport and pH control in fungi. Adv Exp Med Biol 892:33–68. doi:10.1007/978-3-319-25304-6_3. 26721270 PMC5957285

[16] Dechant R, Saad S, Ibáñez AJ, Peter M. 2014. Cytosolic pH regulates cell growth through distinct GTPases, Arf1 and Gtr1, to promote Ras/PKA and TORC1 activity. Mol Cell 55:409–421. doi:10.1016/j.molcel.2014.06.002. 25002144

[17] Dechant R, Binda M, Lee SS, Pelet S, Winderickx J, Peter M. 2010. Cytosolic pH is a second messenger for glucose and regulates the PKA pathway through V-ATPase. EMBO J 29:2515–2526. doi:10.1038/emboj.2010.138. 20581803 PMC2928683

[18] Young BP, Shin JJ, Orij R, Chao JT, Li SC, Guan XL, Khong A, Jan E, Wenk MR, Prinz WA, Smits GJ, Loewen CJ. 2010. Phosphatidic acid is a pH biosensor that links membrane biogenesis to metabolism. Science 329:1085–1088. doi:10.1126/science.1191026. 20798321

[19] Peters LZ, Hazan R, Breker M, Schuldiner M, Ben-Aroya S. 2013. Formation and dissociation of proteasome storage granules are regulated by cytosolic pH. J Cell Biol 201:663–671. doi:10.1083/jcb.201211146. 23690178 PMC3664706

[20] Andrés MT, Acosta-Zaldívar M, González-Seisdedos J, Fierro JF. 2019. Cytosolic acidification is the first transduction signal of lactoferrin-induced regulated cell death pathway. Int J Mol Sci 20:5838. doi:10.3390/ijms20235838. 31757076 PMC6928705

[21] Isom DG, Sridharan V, Baker R, Clement ST, Smalley DM, Dohlman HG. 2013. Protons as second messenger regulators of G protein signaling. Molecular Cell 51:531–538. doi:10.1016/j.molcel.2013.07.012. 23954348 PMC3770139

[22] Miesenböck G, De Angelis DA, Rothman JE. 1998. Visualizing secretion and synaptic transmission with pH-sensitive green fluorescent proteins. Nature 394:192–195. doi:10.1038/28190. 9671304

[23] Kahm M, Navarrete C, Llopis-Torregrosa V, Herrera R, Barreto L, Yenush L, Ariño J, Ramos J, Kschischo M. 2012. Potassium starvation in yeast: mechanisms of homeostasis revealed by mathematical modeling. PLoS Comput Biol 8:e1002548. doi:10.1371/journal.pcbi.1002548. 22737060 PMC3380843

[24] Moskvina E, Imre EM, Ruis H. 1999. Stress factors acting at the level of the plasma membrane induce transcription via the stress response element (STRE) of the yeast Saccharomyces cerevisiae. Mol Microbiol 32:1263–1272. doi:10.1046/j.1365-2958.1999.01438.x. 10383766

[25] Peñalva MA, Tilburn J, Bignell E, Arst HNJ. 2008. Ambient pH gene regulation in fungi: making connections. Trends Microbiol 16:291–300. doi:10.1016/j.tim.2008.03.006. 18457952

[26] Caracuel Z, Roncero MI, Espeso EA, Gonzalez-Verdejo CI, Garcia-Maceira FI, Di Pietro A. 2003. The pH signalling transcription factor PacC controls virulence in the plant pathogen Fusarium oxysporum. Mol Microbiol 48:765–779. doi:10.1046/j.1365-2958.2003.03465.x. 12694620

[27] Niles BJ, Mogri H, Hill A, Vlahakis A, Powers T. 2012. Plasma membrane recruitment and activation of the AGC kinase Ypk1 is mediated by target of rapamycin complex 2 (TORC2) and its effector proteins Slm1 and Slm2. Proc Natl Acad Sci USA 109:1536–1541. doi:10.1073/pnas.1117563109. 22307609 PMC3277121

[28] Niles BJ, Powers T. 2014. TOR complex 2–Ypk1 signaling regulates actin polarization via reactive oxygen species. Mol Biol Cell 25:3962–3972. doi:10.1091/mbc.E14-06-1122. 25253719 PMC4244204

[29] Berchtold D, Piccolis M, Chiaruttini N, Riezman I, Riezman H, Roux A, Walther TC, Loewith R. 2012. Plasma membrane stress induces relocalization of Slm proteins and activation of TORC2 to promote sphingolipid synthesis. Nature Cell Biology 14:542–547. doi:10.1038/ncb2480. 22504275

[30] Martinez-Rocha AL, Roncero MI, Lopez-Ramirez A, Marine M, Guarro J, Martinez-Cadena G, Di Pietro A. 2008. Rho1 has distinct functions in morphogenesis, cell wall biosynthesis and virulence of Fusarium oxysporum. Cell Microbiol 10:1339–1351. doi:10.1111/j.1462-5822.2008.01130.x. 18248628

[31] Casamayor A, Torrance PD, Kobayashi T, Thorner J, Alessi DR. 1999. Functional counterparts of mammalian protein kinases PDK1 and SGK in budding yeast. Curr Biol 9:186–197. doi:10.1016/s0960-9822(99)80088-8. 10074427

[32] García-Marqués S, Randez-Gil F, Dupont S, Garre E, Prieto JA. 2016. Sng1 associates with Nce102 to regulate the yeast Pkh-Ypk signalling module in response to sphingolipid status. Biochim Biophys Acta 1863:1319–1333. doi:10.1016/j.bbamcr.2016.03.025. 27033517

[33] Roelants FM, Breslow DK, Muir A, Weissman JS, Thorner J. 2011. Protein kinase Ypk1 phosphorylates regulatory proteins Orm1 and Orm2 to control sphingolipid homeostasis in Saccharomyces cerevisiae. Proc Natl Acad Sci USA 108:19222–19227. doi:10.1073/pnas.1116948108. 22080611 PMC3228448

[34] Vylkova S. 2017. Environmental pH modulation by pathogenic fungi as a strategy to conquer the host. PLoS Pathog 13:e1006149. doi:10.1371/journal.ppat.1006149. 28231317 PMC5322887

[35] Cruz-Mireles N, Eseola AB, Osés-Ruiz M, Ryder LS, Talbot NJ. 2021. From appressorium to transpressorium-Defining the morphogenetic basis of host cell invasion by the rice blast fungus. PLoS Pathog 17:e1009779. doi:10.1371/journal.ppat.1009779. 34329369 PMC8323886

[36] Morris BM, Reid B, Gow NAR. 1995. Tactic response of zoospores of the fungus Phytophthora palmivora to solutions of different pH in relation to plant infection. Microbiology (Reading) 141:1231–1237. doi:10.1099/13500872-141-5-1231. 33820117

[37] Shaw S, Knüsel S, Abbühl D, Naguleswaran A, Etzensperger R, Benninger M, Roditi I. 2022. Cyclic AMP signalling and glucose metabolism mediate pH taxis by African trypanosomes. Nat Commun 13:603. doi:10.1038/s41467-022-28293-w. 35105902 PMC8807625

[38] Takahashi E, Yamaguchi D, Yamaoka Y. 2020. A relatively small gradient of extracellular pH directs migration of MDA-MB-231 cells in vitro. Int J Mol Sci 21:2565. doi:10.3390/ijms21072565. 32272744 PMC7177698

[39] Yang Y, Sourjik V. 2012. Opposite responses by different chemoreceptors set a tunable preference point in Escherichia coli pH taxis. Mol Microbiol 86:1482–1489. doi:10.1111/mmi.12070. 23078189

[40] Croxen MA, Sisson G, Melano R, Hoffman PS. 2006. The Helicobacter pylori chemotaxis Receptor TlpB (HP0103) Is Required for pH Taxis and for Colonization of the Gastric Mucosa. J Bacteriol 188:2656–2665. doi:10.1128/JB.188.7.2656-2665.2006. 16547053 PMC1428400

[41] Huang JY, Goers Sweeney E, Guillemin K, Amieva MR. 2017. Multiple acid sensors control Helicobacter pylori colonization of the stomach. PLoS Pathog 13:e1006118. doi:10.1371/journal.ppat.1006118. 28103315 PMC5245789

[42] Hesse SJA, Ruijter GJG, Dijkema C, Visser J. 2000. Measurement of intracellular (compartmental) pH by 31P NMR in Aspergillus niger. J Biotechnol 77:5–15. doi:10.1016/S0168-1656(99)00203-5. 10674210

[43] Ullah A, Lopes MI, Brul S, Smits GJ. 2013. Intracellular pH homeostasis in Candida glabrata in infection-associated conditions. Microbiology 159:803–813. doi:10.1099/mic.0.063610-0. 23378571

[44] Rabaste F, Sancelme M, Delort A-M, Blais J, Bolard J. 1995. Intracellular pH of Candida albicans blastospores as measured by laser microspectrofluorimetry and 31P-NMR. Biochim Biophys Acta - Mol Cell Res 1268:41–49. doi:10.1016/0167-4889(95)00042-q. 7626661

[45] Stewart E, Gow NAR, Bowen DV. 1988. Cytoplasmic alkalinization during germ tube formation in Candida albicans. Microbiology 134:1079–1087. doi:10.1099/00221287-134-5-1079. 3058860

[46] Bagar T, Altenbach K, Read ND, Bencina M. 2009. Live-Cell imaging and measurement of intracellular pH in filamentous fungi using a genetically encoded ratiometric probe. Eukaryot Cell 8:703–712. doi:10.1128/EC.00333-08. 19286983 PMC2681602

[47] Srivastava J, Barber DL, Jacobson MP. 2007. Intracellular pH sensors: design principles and functional significance. Physiology 22:30–39. doi:10.1152/physiol.00035.2006. 17289928

[48] Van Drogen F, Dard N, Pelet S, Lee SS, Mishra R, Srejić N, Peter M. 2020. Crosstalk and spatiotemporal regulation between stress-induced MAP kinase pathways and pheromone signaling in budding yeast. Cell Cycle 19:1707–1715. doi:10.1080/15384101.2020.1779469. 32552303 PMC7469626

[49] Brito AS, Neuhäuser B, Wintjens R, Marini AM, Boeckstaens M. 2020. Yeast filamentation signaling is connected to a specific substrate translocation mechanism of the Mep2 transceptor. PLoS Genet 16:e1008634. doi:10.1371/journal.pgen.1008634. 32069286 PMC7048316

[50] Teixeira V, Martins TS, Prinz WA, Costa V. 2021. Target of rapamycin complex 1 (TORC1), protein kinase A (PKA) and cytosolic ph regulate a transcriptional circuit for lipid droplet formation. Int J Mol Sci 22:9017. doi:10.3390/ijms22169017. 34445723 PMC8396576

[51] Lucena RM, Dolz-Edo L, Brul S, de Morais MA, Smits G. 2020. Extreme low cytosolic ph is a signal for cell survival in acid stressed yeast. Genes 11:656. doi:10.3390/genes11060656. 32560106 PMC7349538

[52] Triandafillou CG, Katanski CD, Dinner AR, Drummond DA. 2020. Transient intracellular acidification regulates the core transcriptional heat shock response. Elife 9:e54880. doi:10.7554/eLife.54880. 32762843 PMC7449696

[53] García R, Bravo E, Diez-Muñiz S, Nombela C, Rodríguez-Peña JM, Arroyo J. 2017. A novel connection between the cell wall integrity and the PKA pathways regulates cell wall stress response in yeast. Sci Rep 7:5703. doi:10.1038/s41598-017-06001-9. 28720901 PMC5515849

[54] Wadsworth WG, Riddle DL. 1988. Acidic intracellular pH shift during Caenorhabditis elegans larval development. Proc Natl Acad Sci USA 85:8435–8438. doi:10.1073/pnas.85.22.8435. 3186732 PMC282472

[55] Rathje M, Fang H, Bachman JL, Anggono V, Gether U, Huganir RL, Madsen KL. 2013. AMPA receptor pHluorin-GluA2 reports NMDA receptor-induced intracellular acidification in hippocampal neurons. Proc Natl Acad Sci USA 110:14426–14431. doi:10.1073/pnas.1312982110. 23940334 PMC3761605

[56] Salsaa M, Aziz K, Lazcano P, Schmidtke MW, Tarsio M, Hüttemann M, Reynolds CA, Kane PM, Greenberg ML. 2021. Valproate activates the Snf1 kinase in Saccharomyces cerevisiae by decreasing the cytosolic pH. J Biol Chem 297:101110. doi:10.1016/j.jbc.2021.101110. 34428448 PMC8449051

[57] Henderson KA, Hughes AL, Gottschling DE. 2014. Mother-daughter asymmetry of pH underlies aging and rejuvenation in yeast. Elife 3:e03504. doi:10.7554/eLife.03504. 25190112 PMC4175738

[58] Rane HS, Hayek SR, Frye JE, Abeyta EL, Bernardo SM, Parra KJ, Lee SA. 2019. Candida albicans Pma1p contributes to growth, pH homeostasis, and hyphal formation. Front Microbiol 10:1012. doi:10.3389/fmicb.2019.01012. 31143168 PMC6521590

[59] Li J, Yang S, Li D, Peng L, Fan G, Pan S. 2022. The plasma membrane H+-ATPase is critical for cell growth and pathogenicity in Penicillium digitatum. Appl Microbiol Biotechnol 106:5123–5136. doi:10.1007/s00253-022-12036-4. 35771244

[60] Mira NP, Palma M, Guerreiro JF, Sá-Correia I. 2010. Genome-wide identification of Saccharomyces cerevisiae genes required for tolerance to acetic acid. Microb Cell Fact 9:79. doi:10.1186/1475-2859-9-79. 20973990 PMC2972246

[61] Guerreiro JF, Muir A, Ramachandran S, Thorner J, Sa-Correia I. 2016. Sphingolipid biosynthesis upregulation by TOR complex 2-Ypk1 signaling during yeast adaptive response to acetic acid stress. Biochem J 473:4311–4325. doi:10.1042/BCJ20160565. 27671892 PMC5124397

[62] Colabardini AC, Brown NA, Savoldi M, Goldman MH, Goldman GH. 2013. Functional characterization of Aspergillus nidulans ypkA, a homologue of the mammalian kinase SGK. PLoS One 8:e57630. doi:10.1371/journal.pone.0057630. 23472095 PMC3589345

[63] Fabri JHTM, Godoy NL, Rocha MC, Munshi M, Cocio TA, von Zeska Kress MR, Fill TP, da Cunha AF, Del Poeta M, Malavazi I. 2019. The AGC kinase YpkA regulates sphingolipids biosynthesis and physically interacts with SakA MAP kinase in Aspergillus fumigatus. Front Microbiol 9:3347. doi:10.3389/fmicb.2018.03347. 30692984 PMC6339957

[64] Tani M. 2016. Function Relationship of Complex Sphingolipids in Yeast. Trends Glycosci Glycotechnol 28:E109–E116. doi:10.4052/tigg.1509.1E.

[65] Liu K, Zhang X, Lester RL, Dickson RC. 2005. The sphingoid long chain base phytosphingosine activates AGC-type protein kinases in Saccharomyces cerevisiae including Ypk1, Ypk2, and Sch9. J Biol Chem 280:22679–22687. doi:10.1074/jbc.M502972200. 15840588

[66] Roelants FM, Torrance PD, Bezman N, Thorner J. 2002. Pkh1 and Pkh2 differentially phosphorylate and activate Ypk1 and Ykr2 and define protein kinase modules required for maintenance of cell wall integrity. Mol Biol Cell 13:3005–3028. doi:10.1091/mbc.e02-04-0201. 12221112 PMC124139

[67] Schmelzle T, Helliwell SB, Hall MN. 2002. Yeast protein kinases and the RHO1 exchange factor TUS1 are novel components of the cell integrity pathway in yeast. Mol Cell Biol 22:1329–1339. doi:10.1128/MCB.22.5.1329-1339.2002. 11839800 PMC134704

[68] Lindberg L, Santos AXS, Riezman H, Olsson L, Bettiga M. 2013. Lipidomic Profiling of Saccharomyces cerevisiae and Zygosaccharomyces bailii Reveals Critical Changes in Lipid Composition in Response to Acetic Acid Stress. PLoS One 8:e73936. doi:10.1371/journal.pone.0073936. 24023914 PMC3762712

[69] Jenkins GM, Richards A, Wahl T, Mao C, Obeid L, Hannun Y. 1997. Involvement of yeast sphingolipids in the heat stress response of Saccharomyces cerevisiae. J Biol Chem 272:32566–32572. doi:10.1074/jbc.272.51.32566. 9405471

[70] He Y, Li D, Cook SL, Yoon M-S, Kapoor A, Rao CV, Kenis PJA, Chen J, Wang F. 2013. Mammalian target of rapamycin and Rictor control neutrophil chemotaxis by regulating Rac/Cdc42 activity and the actin cytoskeleton. Mol Biol Cell 24:3369–3380. doi:10.1091/mbc.E13-07-0405. 24006489 PMC3814157

[71] Ikeda M, Kihara A, Denpoh A, Igarashi Y. 2008. The Rim101 pathway is involved in rsb1 expression induced by altered lipid asymmetry. Mol Biol Cell 19:1922–1931. doi:10.1091/mbc.e07-08-0806. 18287536 PMC2366876

[72] Nishino K, Obara K, Kihara A. 2015. The C-terminal cytosolic region of Rim21 senses alterations in plasma membrane lipid composition: insights into sensing mechanisms for plasma membrane lipid asymmetry. J Biol Chem 290:30797–30805. doi:10.1074/jbc.M115.674382. 26527678 PMC4692209

[73] Obara K, Kamura T. 2021. The Rim101 pathway mediates adaptation to external alkalization and altered lipid asymmetry: hypothesis describing the detection of distinct stresses by the Rim21 sensor protein. Curr Genet 67:213–218. doi:10.1007/s00294-020-01129-0. 33184698

[74] Munder MC, Midtvedt D, Franzmann T, Nüske E, Otto O, Herbig M, Ulbricht E, Müller P, Taubenberger A, Maharana S, Malinovska L, Richter D, Guck J, Zaburdaev V, Alberti S. 2016. A pH-driven transition of the cytoplasm from a fluid- to a solid-like state promotes entry into dormancy. Elife 5:e09347. doi:10.7554/eLife.09347. 27003292 PMC4850707

[75] López-Berges MS, Capilla J, Turrà D, Schafferer L, Matthijs S, Jöchl C, Cornelis P, Guarro J, Haas H, Di Pietro A. 2012. HapX-Mediated Iron Homeostasis Is Essential for Rhizosphere Competence and Virulence of the Soilborne Pathogen Fusarium oxysporum. Plant Cell 24:3805–3822. doi:10.1105/tpc.112.098624. 22968717 PMC3480304

[76] Ozaki K, Tanaka K, Imamura H, Hihara T, Kameyama T, Nonaka H, Hirano H, Matsuura Y, Takai Y. 1996. Rom1p and Rom2p are GDP/GTP exchange proteins (GEPs) for the Rho1p small GTP binding protein in Saccharomyces cerevisiae. EMBO J 15:2196–2207. doi:10.1002/j.1460-2075.1996.tb00573.x. 8641285 PMC450143

[77] Méchin V, Damerval C, Zivy M. 2007. Total protein extraction with TCA-acetone. Methods Mol Biol 355:1–8. 17093296 10.1385/1-59745-227-0:1

[78] Punt PJ, Oliver RP, Dingemanse MA, Pouwels PH, van den Hondel CA. 1987. Transformation of Aspergillus based on the hygromycin B resistance marker from Escherichia coli. Gene 56:117–124. doi:10.1016/0378-1119(87)90164-8. 2824287

[79] Fernandes TR, Serrano A, Di Pietro A. 2022. In Vivo Monitoring of Cytosolic pH Using the Ratiometric pH Sensor pHluorin. Methods Mol Biol 2391:99–107. doi:10.1007/978-1-0716-1795-3_9. 34686980

[80] Bielawski J, Pierce JS, Snider J, Rembiesa B, Szulc ZM, Bielawska A. 2010. Sphingolipid Analysis by High Performance Liquid Chromatography-Tandem Mass Spectrometry (HPLC-MS/MS), p 46–59. *In* Chalfant C, Poeta MD (ed), Sphingolipids as Signaling and Regulatory Molecules. Springer New York, New York, NY. doi:10.1007/978-1-4419-6741-1_3. 20919645

