# Supplementary material for: Cytosolic pH Controls Fungal MAPK Signaling and Pathogenicity
Source: mBio. 2023 Mar 2;14(2):e00285-23. doi: 10.1128/mbio.00285-23 (PMC10128062; doi:10.1128/mbio.00285-23)
Supplement: FIG S1 [file mbio.00285-23-s0001.pdf]

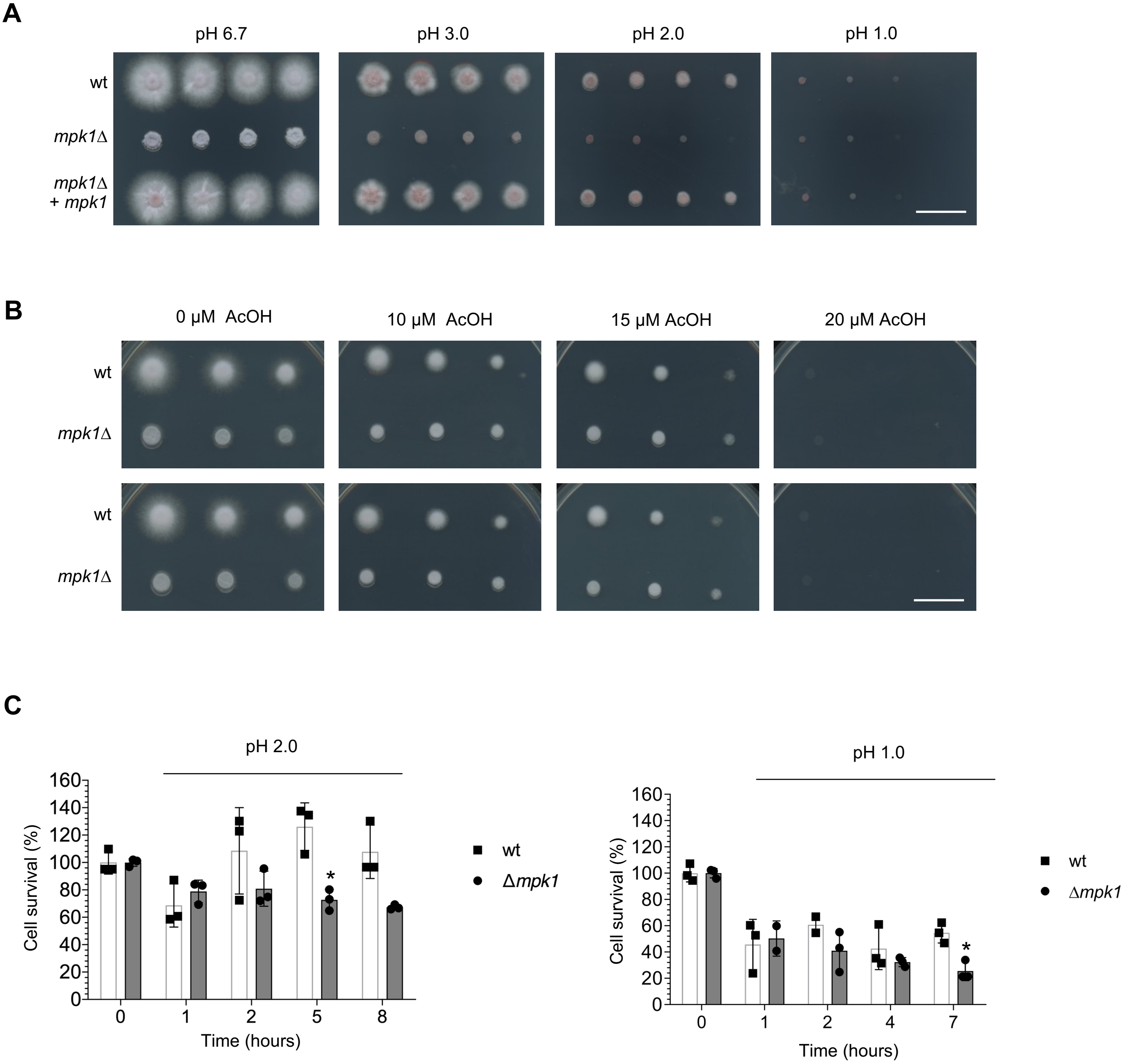

FIG S1 The CWI MAPK Mpk1 has a minor role in adaptation to acidic pH.

A,B) Serial dilutions of fresh microconidia of the indicated strains were spot-inoculated on PDA plates adjusted to the indicated pH values by adding HCl (A) or supplemented with the indicated concentrations of acetic acid (AcOH) (B). Plates were incubated at 28ºC in the dark and imaged after 3 days. Images shown are representative of two independent experiments with three plates each. Scale bar, 2 cm. C) The percentage of cell survival of the *F. oxysporum* wild type (wt) and the *mpk1*Δ mutant after the indicated times of exposure to KSU buffer adjusted to pH 2 or 1 by adding HCl was measured by dilution plating and colony counting and normalized to time 0. * $p<0.05$ *versus* wt according to Welch's t-test. Data show the mean ± s.d. of three replicate microwells.
