# Supplementary material for: Cytosolic pH Controls Fungal MAPK Signaling and Pathogenicity
Source: mBio. 2023 Mar 2;14(2):e00285-23. doi: 10.1128/mbio.00285-23 (PMC10128062; doi:10.1128/mbio.00285-23)
Supplement: FIG S2 [file mbio.00285-23-s0002.pdf]

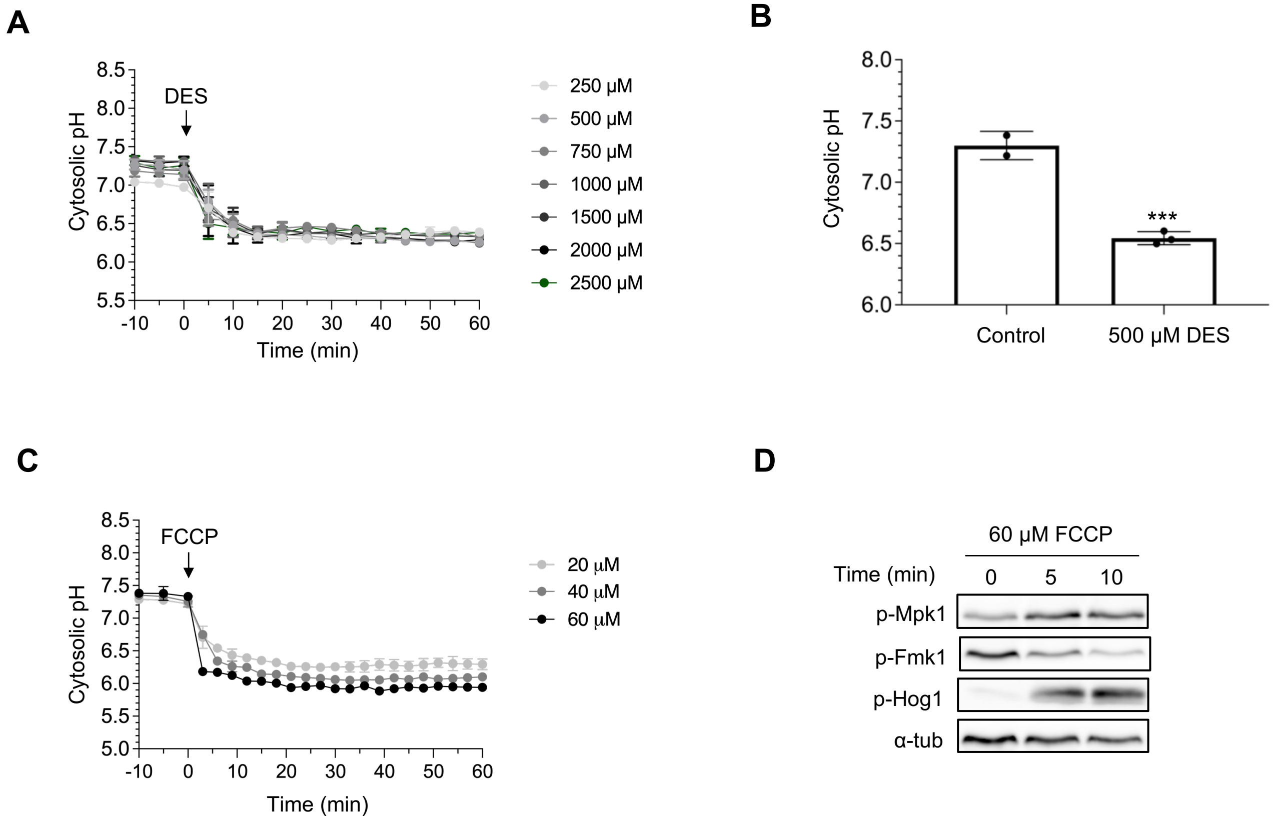

FIG S2 Pma1 inhibition by DES or membrane depolarization causes rapid acidification of pH_c. A,B) Pma1 inhibition by DES causes rapid and sustained acidification of pH_c. *F. oxysporum* microconidia were pretreated as described in Fig. 3 before adding the indicated concentrations of DES to the medium. pH_c was monitored spectrofluorometrically (A) or by confocal microscopy (B) as described in Fig. 3 (C) or (B), respectively. Data show the mean ± s.d. of three independent replicate microwells from one representative experiment. Experiments were performed twice with similar results. C) Membrane depolarization by carbonyl cyanide p-trifluoromethoxyphenylhydrazone (FCCP) causes rapid and sustained acidification of pH_c. *F. oxysporum* microconidia were pretreated as described in Fig. 3 before adding the indicated concentrations of FCCP to the medium. pH_c was monitored spectrofluorometrically as described in Fig. 3. Data show the mean ± s.d. of three independent replicate microwells from one representative experiment. Experiments were performed twice with similar results. D) *F. oxysporum* microconidia were pretreated as described in Fig. 3, and 60 µM FCCP was added to the medium. Total protein extracts collected at the indicated times were subjected to immunoblot analysis with different antibodies as indicated in Fig. 2.
