# Supplementary material for: Cytosolic pH Controls Fungal MAPK Signaling and Pathogenicity
Source: mBio. 2023 Mar 2;14(2):e00285-23. doi: 10.1128/mbio.00285-23 (PMC10128062; doi:10.1128/mbio.00285-23)
Supplement: FIG S3 [file mbio.00285-23-s0003.pdf]

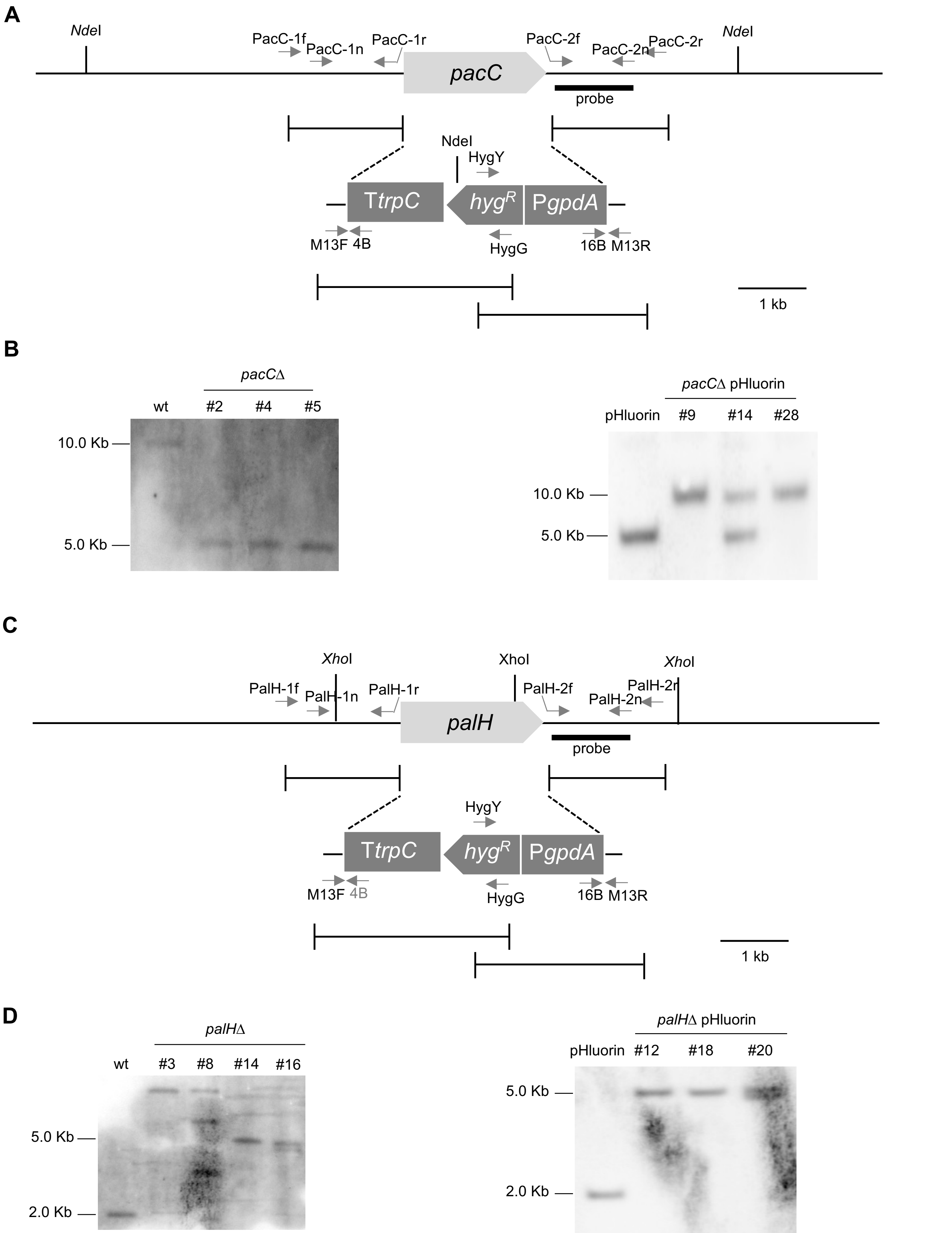

FIG S3 Targeted deletion of *pacC* and *palH* in *F. oxysporum*.
A,C) Schematic diagram showing targeted deletion of the *F. oxysporum pacC* (A) and *palH* (C) genes using the split-marker method. Gene knockout constructs were obtained by fusion PCR. Relative positions of restriction sites and Southern probes as well as of the PCR primers used are indicated. *hygR*, hygromycin resistance gene; P*gpdA*, *gpdA* promoter; T*trpC*, *trpC* terminator (both from *A. nidulans*). B,D) Genomic DNA of independent transformants obtained in the wild type (wt, left panels) or the pHluorin-expressing background (right panels) was treated with *Nde*I (B) or *Xho*I (D), separated on 0.7% agarose gels, transferred to nylon membranes and hybridized with DIG labelled DNA probes from the indicated genes. Molecular weights of the hybridizing bands are indicated on the left.
