# Supplementary material for: Cytosolic pH Controls Fungal MAPK Signaling and Pathogenicity
Source: mBio. 2023 Mar 2;14(2):e00285-23. doi: 10.1128/mbio.00285-23 (PMC10128062; doi:10.1128/mbio.00285-23)
Supplement: FIG S4 [file mbio.00285-23-s0004.pdf]

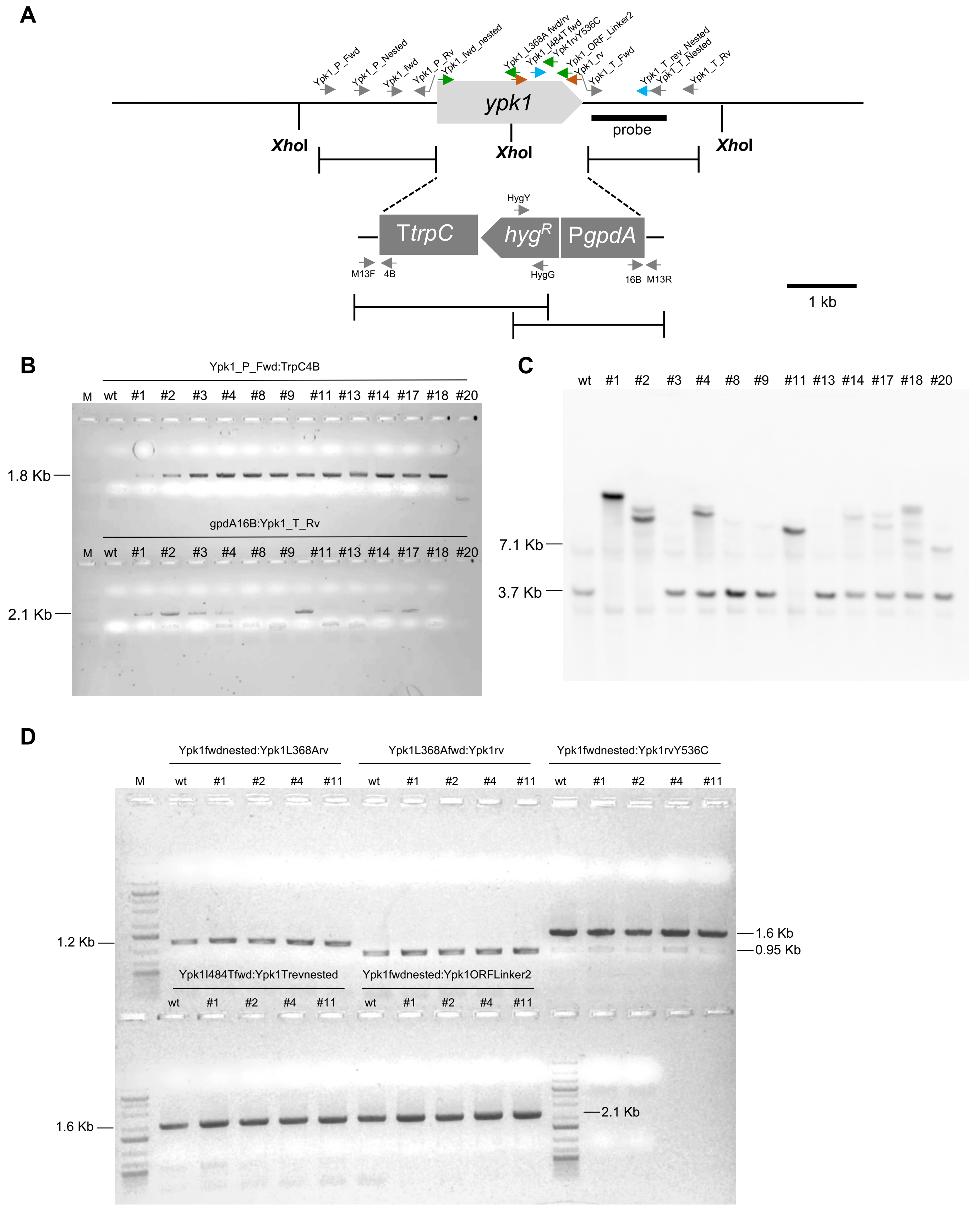

FIG S4 Failure to obtain *ypk1*Δ knockout mutants suggests that Ypk1 is essential in *F. oxysporum*.

A) Schematic diagram showing the targeted deletion of the *F. oxysporum ypk1* gene using the split-marker method. Gene knockout constructs were obtained by fusion PCR. Relative positions of restriction sites and Southern probes as well as of the PCR primers are indicated. Combinations of primers with the same colour were used for the PCR analyses in (C) and (D). *hygR*, hygromycin resistance gene; P*gpdA*, *gpdA* promoter; T*trpC*, *trpC* terminator (both from *A. nidulans*). B,C) Genomic DNA of the wild type (wt) and independent hygromycin resistant transformants was subjected to PCR with the indicated pairs of primers (B) or treated with *Xho*I (C). The samples were separated on 0.7% agarose gels and imaged (B) or transferred to nylon membranes and hybridized with the DIG labelled DNA probe (C). Relative positions of the expected wild type or knockout (KO) hybridizing bands in (C) are indicated on the left. D) Genomic DNA of the wild type (wt) and independent hygromycin resistant transformants was subjected to PCR with the indicated pairs of primers, separated on 0.7% agarose gels and imaged. M, Molecular size markers.
