# Supplementary material for: Cytosolic pH Controls Fungal MAPK Signaling and Pathogenicity
Source: mBio. 2023 Mar 2;14(2):e00285-23. doi: 10.1128/mbio.00285-23 (PMC10128062; doi:10.1128/mbio.00285-23)
Supplement: FIG S5 [file mbio.00285-23-s0005.pdf]

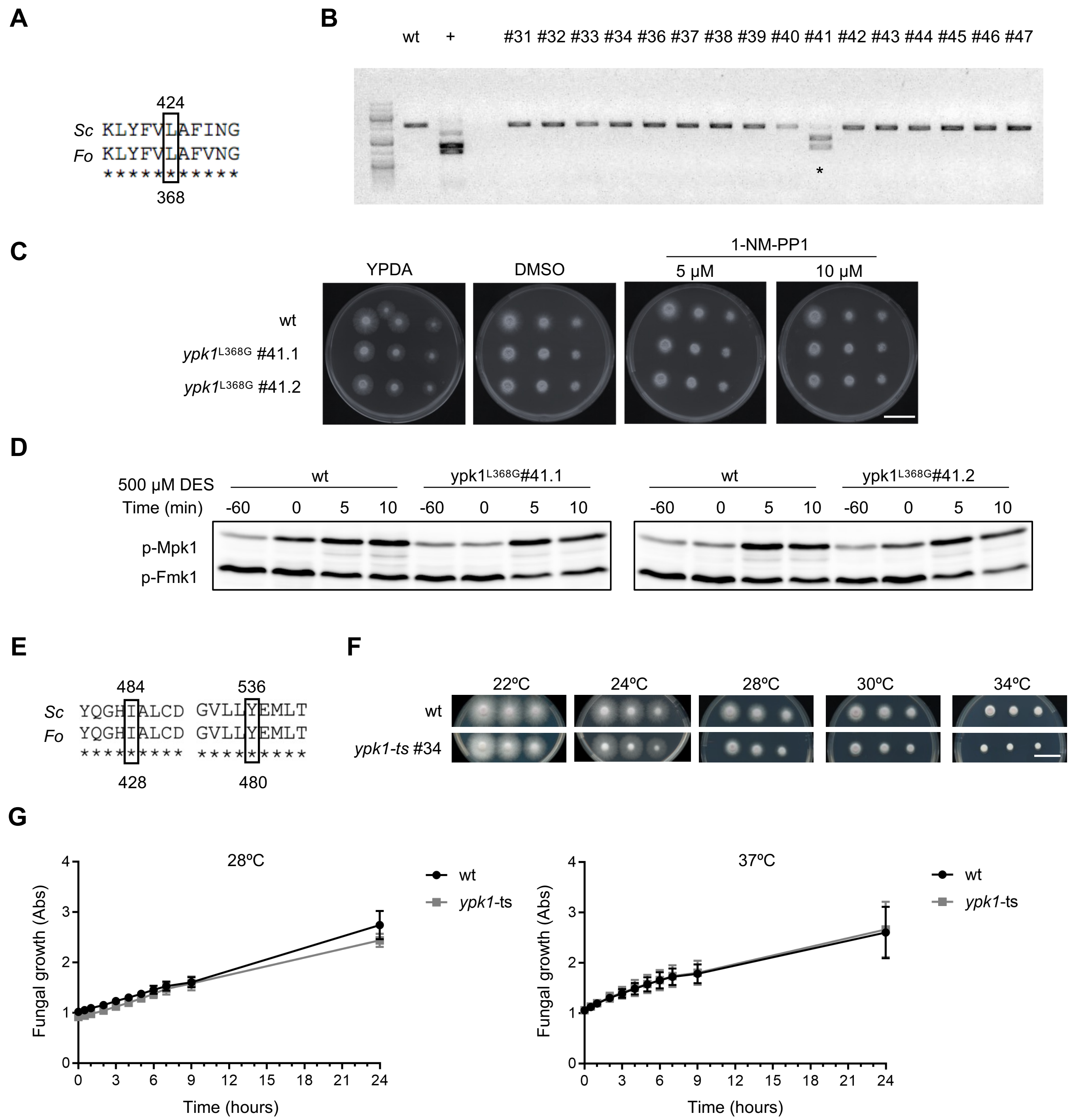

FIG S5 Attempts of mutational analysis of *ypk1* in *F. oxysporum*.

A) Amino acid alignment showing the contextual conservation in *F. oxysporum* of the L424 residue of *S. cerevisiae* Ypk1, whose mutation to G in yeast causes sensitivity to the analog 1-NM-PP1 (Berchtold *et al*., 2012). B) Screening of *F. oxysporum* transformants for the L368G mutation using RFLP analysis. Genomic DNA of the wild type (wt) and independent hygromycin resistant transformants was subjected to PCR followed by treatment with the restriction enzyme *Nar*I which cuts the DNA fragment carrying the *ypk1*^{L368G} mutation. As a positive control (+), PCR was performed on the DNA construct employed for transformation. C) Serial dilutions of fresh microconidia of the wt and two monoconidial isolates of transformant #41 carrying the *ypk1*^{L368G} mutation were spot-inoculated on plates containing YPDA supplemented with the indicated concentrations of 1-NM-PP1. Plates were incubated at 28ºC in the dark and imaged after 2 days. Images shown are representative of three independent biologic replicates. Scale bar, 2 cm. D) Western blot showing MAPK phosphorylation in response to 500 µM DES in the wt and two monoconidial isolates of transformant #41 carrying the *ypk1*^{L368G} mutation. The specific Ypk1-AS inhibitor 1-NM-PP1 (40 µM) was added 60 min before DES addition (-60). Protein extracts were subjected to immunoblot analysis with anti-phospho-p44/42 MAPK antibody to detect phosphorylated p-Mpk1 and p-Fmk1. E) Amino acid alignment showing the contextual conservation in *F. oxysporum* of the I484 and Y536 residues of *S. cerevisiae* Ypk1, whose simultaneous mutation to T and C, respectively, causes temperature sensitivity in yeast. F) Analysis of temperature sensitivity in the *F. oxysporum* wild type strain and *ypk1-ts* #34 transformant carrying the I428T and Y480C mutations. Serial dilutions of fresh microconidia were spot-inoculated on PDA plates, incubated at indicated temperatures in the dark and imaged after 3 days. Images shown are representative of three independent biologic replicates. Scale bar, 2 cm. G) Growth of the wt and the y*pk1*-ts #34 strain in PDB at 28ºC or 37ºC was monitored by measuring absorbance (Abs) at 600 nm. Values were normalized to time zero. Data show the mean ± s.d. of three independent replicates from one representative experiment. Experiments were performed twice with similar results.
