# Supplementary material for: Cytosolic pH Controls Fungal MAPK Signaling and Pathogenicity
Source: mBio. 2023 Mar 2;14(2):e00285-23. doi: 10.1128/mbio.00285-23 (PMC10128062; doi:10.1128/mbio.00285-23)
Supplement: FIG S6 [file mbio.00285-23-s0006.pdf]

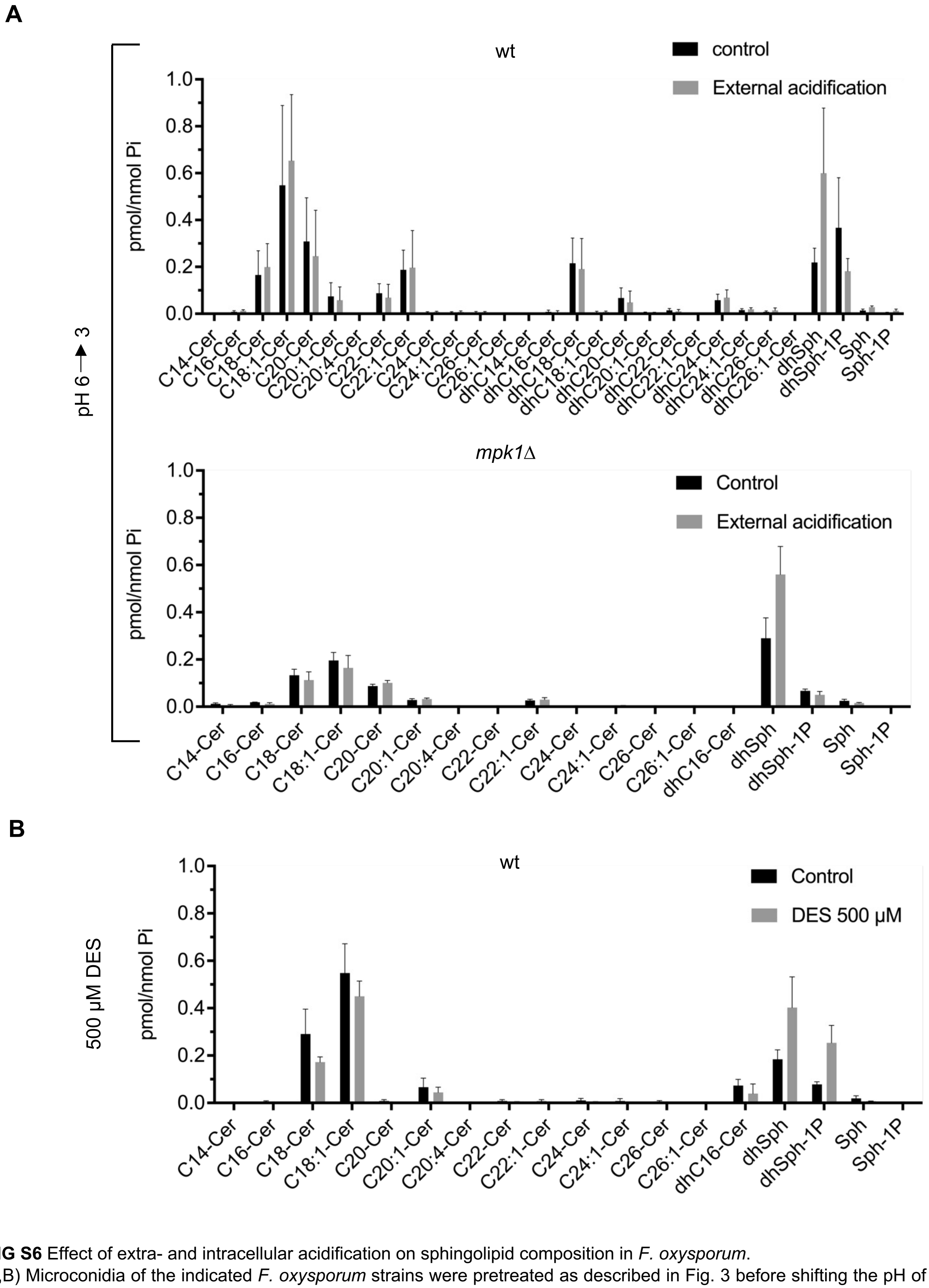

FIG S6 Effect of extra- and intracellular acidification on sphingolipid composition in *F. oxysporum*.

A,B) Microconidia of the indicated *F. oxysporum* strains were pretreated as described in Fig. 3 before shifting the pH of the medium from 6 to 3 (A) or adding 500 µM DES (B). Samples were collected before (control) or 10 minutes after treatment. Extracted lipids were analyzed by HPLC/MS-MS and the concentration of each ceramide molecular species was normalized to total phosphate levels (Pi). Each graph shows the mean ± s.d. of three independent experiments.
