# Supplementary material for: Cytosolic pH Controls Fungal MAPK Signaling and Pathogenicity
Source: mBio. 2023 Mar 2;14(2):e00285-23. doi: 10.1128/mbio.00285-23 (PMC10128062; doi:10.1128/mbio.00285-23)
Supplement: TABLE S1 [file mbio.00285-23-s0007.pdf]

Table S1. *Fusarium oxysporum* strains used in this study.

**Strain**	**Genotype**	**Reference**
FGSC 4287	Wild type	(Di Pietro *et al.*, 2001)
*bck1*Δ	*bck1*::*HYG*	(Turrà *et al.*, 2015)
*fmk1*Δ	*fmk1*::*PHLEO*	(Di Pietro *et al.*, 2001)
*fmk1*Δ *hog1*Δ	*fmk1*::*PHLEO; hog1*::*HYG*	(Segorbe *et al.*, 2017)
*fmk1*Δ+*fmk1*	*fmk1*::*PHLEO*; *fmk1*+*HYG*	(Di Pietro *et al.*, 2001)
*hog1*Δ	*hog1*::*HYG*	(Segorbe *et al.*, 2017)
*hog1*Δ+*hog1*	*hog1*::*HYG*; *hog1*+*PHLEO*	(Segorbe *et al.*, 2017)
*mpk1*Δ	*mpk1*::*HYG*	(Turrà *et al.*, 2015)
*mpk1*Δ *fmk1*Δ	*mpk1*::*HYG; fmk1*::*PHLEO*	(Turrà *et al.*, 2015)
mpk1Δ hog1Δ	*mpk1*::*HYG; hog1*::*PHLEO*	(Segorbe *et al.*, 2017)
*mpk1*Δ+*mpk1*	*mpk1*::*HYG*; *mpk1*+*PHLEO*	(Turrà *et al.*, 2015)
*pacC*Δ	*pacC*::*HYG*	This study
*pacC*Δ::*pHluorin*	*pHluorin*; *HYG*; *pacC*::NEO	This study
*palH*Δ	*palH*::*HYG*	This study
*palH*Δ::p*Hluorin*	*pHluorin*; *HYG*; *paLH*::NEO	This study
pHluorin	*pHluorin*; *HYG*	(Fernandes *et al.*, 2022)
*rho1*Δ	*rho1*::*HYG*	(Martinez-Rocha *et al.*, 2008)
