# Supplementary material for: Cytosolic pH Controls Fungal MAPK Signaling and Pathogenicity
Source: mBio. 2023 Mar 2;14(2):e00285-23. doi: 10.1128/mbio.00285-23 (PMC10128062; doi:10.1128/mbio.00285-23)
Supplement: TABLE S2 [file mbio.00285-23-s0008.pdf]

Table S2. *Saccharomyces cerevisiae* strains used in this study.

**Strain**	**Gentoype**	**Reference**
BY4741	*MATa his3*Δ1 *leu2*Δ0 *met15*Δ0 *ura3*Δ0	Euroscarf
pHluorin	BY4741; pYEplac181-Tef1-pHluorin	(Isom *et al.*, 2013)
*bck1*Δ	BY4741 *bck1::KanMX4*	Euroscarf
*mid2*Δ	BY4741 *mid2::KanMX4*	Euroscarf
*mtl1*Δ	BY4741 *mtl1::KanMX4*	Euroscarf
*pkh1*Δ	BY4741 *pkh1::KanMX4*	Euroscarf
*pkh2*Δ	BY4741 *pkh2::KanMX4*	Euroscarf
*rom2*Δ	BY4741 *rom2::KanMX4*	Euroscarf
*wsc1*Δ	BY4741 *wsc1::KanMX4*	Euroscarf
*ypk1*Δ	BY4741 *ypk1::KanMX4*	Euroscarf
*ypk2*Δ	BY4741 *ypk2::KanMX4*	Euroscarf
YPH499	*MATa ade2-10 trp1-63 leu2-1 ura3-52 his3-Δ200 lys2-801*	(Sikorski and Hieter, 1989)
*ypk1-ts/ ypk2*Δ	YPH499 *ypk1-ts::HIS3 ypk2::TRP1*	(Casamayor *et al.*, 1999)
OHNY	*MATa ura3 his3 trp1 leu2 ade2*	(Ozaki *et al.*, 1996)
rho1-ts	OHNY *rho1–104*	(Ozaki *et al.*, 1996)
SEY6221	*MATa leu2-3,112 ura3-52 his3Δ200 trp1Δ901 suc2Δ9 ade2-101*	(Paravicini *et al.*, 1992)
pkc1-ts	SEY6221 *pkc1Δ1::HIS3*	(Paravicini *et al.*, 1992)
TB50a	MATa *trp1 his3 ura3 leu2*	(Berchtold *et al.*, 2012)
ypk1^{L424G} *ypk2*Δ	TB50a *ypk1*Δ::*KANMX4 ypk2*Δ::*HIS3* pRS416-ypk1^{L424G}	(Berchtold *et al.*, 2012)
